# Sodium Silicate Grouting: Mechanisms, Environmental Impacts, and Research Directions

**DOI:** 10.1007/s11242-026-02323-3

**Published:** 2026-06-19

**Authors:** Mohammad Valibeknejad, Thomas Sweijen, Alraune Zech, Julian Quodbach, Noushine Shahidzadeh, Mariette Wolthers, Amir Raoof

**Affiliations:** 1 Environmental Hydrogeology Group, Department of Earth Sciences, Utrecht University, Utrecht, The Netherlands; 2 Division of Pharmaceutics, Utrecht Institute for Pharmaceutical Sciences, Utrecht University, Utrecht, The Netherlands; 3 Institute of Physics, University of Amsterdam, Amsterdam, The Netherlands; 4 Geochemistry Group, Department of Earth Sciences, Utrecht University, Utrecht, The Netherlands

**Keywords:** Sodium silicate grouting, Permeability reduction, Soil reinforcement, Gelation mechanisms, Gel degradation, Environmental impact

## Abstract

Sodium silicate grouting is a widely used technique for reducing soil permeability and reinforcing unconsolidated soils. Despite decades of use, its field performance remains inconsistent, with efficiency strongly influenced by soil heterogeneity, groundwater composition, and contaminant interactions. In addition, the environmental risks associated with silica gels, such as leaching of diluted grout into groundwater and long-term degradation, are not fully understood. This review, the first comprehensive synthesis since the 1990s, integrates insights from scientific studies, field applications, and industry practice. We examine the coupled processes governing injection, gelation, and post-gelation evolution across micro-, pore-, and field scales, and identify the key hydraulic, chemical, thermal, and environmental parameters controlling grouting performance. Particular attention is given to sand–gel interactions, microstructural development, permeability reduction mechanisms, and factors governing strength enhancement. The review also evaluates environmental impact and discusses the potential of Electrical Resistivity Tomography (ERT) for monitoring grout continuity. Finally, we outline critical research directions needed to develop constitutive relationships for grout properties, improve predictive modelling, and enable more reliable, sustainable, and environmentally compatible applications of silicate grouting.

**Supplementary Information:**

The online version contains supplementary material available at 10.1007/s11242-026-02323-3.

## Article Highlights

- Environmental impacts of silica gel on groundwater.
- Effect of groundwater contamination on grouting efficiency.
- Processes during injection and gel formation as well as presence stage of the grouting.

**Supplementary Information:**

The online version contains supplementary material available at 10.1007/s11242-026-02323-3.

## Introduction

### Grouting for Permeability Reduction and Soil Reinforcement

Urbanization in deltaic and coastal regions with unconsolidated subsurface deposits has driven an increasing demand for underground infrastructure, including transport systems, parking facilities, and drainage networks. Construction in these environments typically requires deep excavations supported by soil-retaining walls, where groundwater control is a critical challenge (Hack et al. 2004; Lin et al. 2016; Littlejohn et al. 1997; Luger et al. 2022; Mollamahmutoğlu and Avci 2016; Powers et al. 2007; Pujades et al. 2014). In sandy and gravelly soils, conventional dewatering can induce excessive drawdowns, leading to soil settlement, displacement of contaminants (Aurand et al. 1981; Elektorowicz et al. 2008; Fetter et al. 2017; Spacagna et al. 2017), and adverse impacts on shallow aquifers that are often used for geothermal energy storage (Bloemendal and Hartog 2018; Martínez-León et al. 2025).

Grouting provides an alternative by sealing aquifers beneath construction pits, thereby reducing inflow and limiting the need for large-scale dewatering. This technique forms temporary, low-permeability barriers within designated sand layers and is often used in combination with limited pumping to achieve stable, dry working conditions. Beyond permeability control, grouting is also applied to improve soil strength in weak, unconsolidated sands with large pores, low cementation, and poor bearing capacity (Fabozzi et al. 2020; Güllü et al. 2023; Sha et al. 2024). In such cases, permeation grouting reinforces the soil structure without disturbing it, as the grout penetrates under low pressure and binds particles into a solid framework (Elipe and López-Querol 2014).

The choice of grout depends on soil properties (grain size, porosity, and permeability) and environmental conditions (temperature, groundwater chemistry, contamination, and organic matter). Conventional cement suspensions (Chupin et al. 2007; Zhou et al. 2018) and Bentonite based grouts (Laviña et al. 2023; Yoon et al. 2014) are effective in coarse soils but are filtered out in finer sands. Chemical grouts, by contrast, can permeate smaller pores due to their initially low viscosity and controlled gelation (Wang et al. 2023c). A wide range of chemical grouts has been developed, including acrylic acids (Rosewitz et al. 2016), aminoplast (Funehag and Gustafson 2008), phenoplast (Funehag and Gustafson 2008), epoxy resins (Santarato et al. 2011; Zhang et al. 2019), acrylate (Wang et al. 2023b), urethane (Black 1977; Saleh et al. 2019), tetraethoxysilane (Maravelaki-Kalaitzaki et al. 2008), acrylamide (Ozgurel and Vipulanandan 2005), colloidal silica (Bolisetti et al. 2009; Gallagher et al. 2007; Krishnan and Shukla 2021), and hybrid formulations (Park and Oh 2018; Pedrotti et al. 2020; Seiphoori and Zamanian 2022). More recently, microbial-induced carbonate precipitation (MICP) (Barkouki et al. 2011; Hommel et al. 2015; Konstantinou et al. 2023) and Biofilm growth (Hommel et al. 2018; Jung and Meile 2021) has attracted attention for sustainable soil stabilization, although high costs remain a limitation (Xu et al. 2022). Among these options, sodium silicate grouts (commonly referred to as waterglass) stand out due to their favorable injectability, low cost, established safety record, and relatively benign environmental profile (Bodocsi and Bowers 1991; Krizek and Spino 2000; Navarro-Moreno et al. 2021; US Army Corps of Engineers 1995; Wang et al. 2023c; Xu et al. 2023; Zhu et al. 2021; Zullo et al. 2020).

### Broader Application of Sodium Silicate

Beyond its role in soil stabilization, sodium silicate has been applied across diverse engineering and environmental domains. In subsurface and reservoir engineering, it has been used for water management in fractured carbonate formations (Hatzignatiou and Giske 2018), fluid control in reservoirs (Hashemi et al. 2023), and wellbore sealing during well operations (Liu and Ott 2020). In construction materials, sodium silicate has been incorporated to regulate hydration and setting in ultra-high-performance concretes (Abdalla et al. 2022; Alrousan and Alnemrawi 2022; Khan et al. 2023), as an additive for ultrafine slag-based cement to enhance strength and reduce permeability (Avci et al. 2021), mixed with aluminum sulfate to stabilize high liquid limit clay subgrades (Zhang et al. 2015), combined with other chemical grouts to improve flexibility (Yang et al. 2017) and water-blocking efficiency (Wang et al. 2023a). Its chemical versatility has also enabled applications in challenging environments, including submarine tunnel construction (Li et al. 2021), encapsulation of contaminated aquifers (Bodocsi and Bowers 1991; Elektorowicz et al. 2008), neutralization of acidic zones affected by mining (Anagnostopoulos et al. 2017), stabilization of sulfate-bearing soil (Li et al. 2023), and strengthening of marine clays (Pakir et al. 2015). In environmental engineering, sodium silicate has been explored for CO_2_ capture (Do et al. 2024; Liu et al. 2024), methane drainage in coal seams (Lu et al. 2018), wastewater treatment (Wright et al. 2025), heavy metal removal (Nyirenda et al. 2022), and stabilization of salt formations and brine reservoirs (Engelhardt 2014). These examples underscore the adaptability of sodium silicate across disciplines. Building on this broad foundation, the present review focuses specifically on the application of sodium silicate grouting in geotechnical engineering, referred to hereafter as silicate grouting, for enhancing soil strength and reducing permeability (Hurley and Thornburn 1971).

### Conceptual Model of Silicate Grouting

**Fig. 1:**
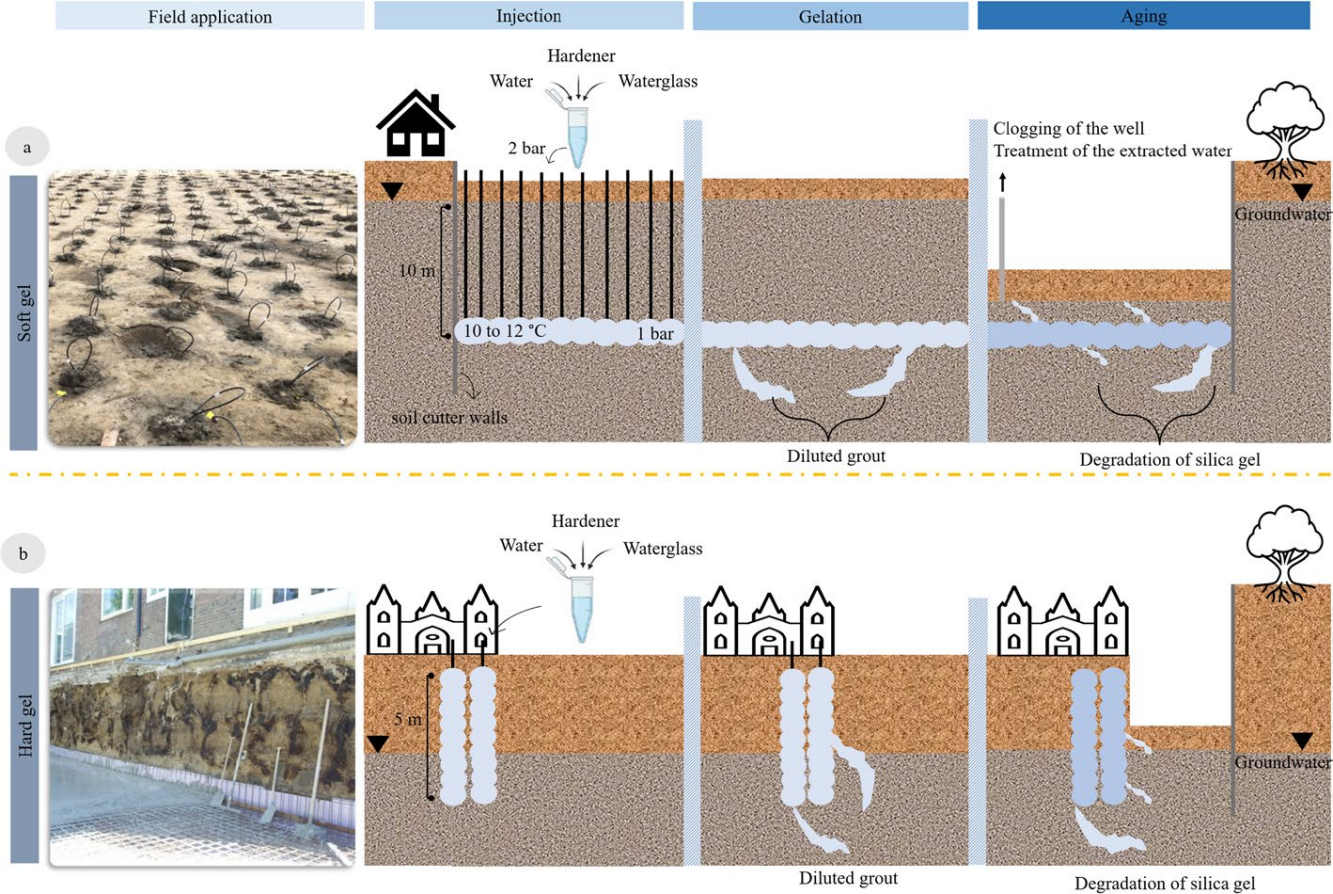
Silicate Grouting: Schematic illustration of silicate grout application in soils, showing two main functions: **a** creation of a low-permeability barrier with soft gels to enable dry construction pits, and **b** soil reinforcement with hard gels to improve foundation stability. The process involves three stages: (i) injection stage, where grout is introduced into the soil through tubes installed at specific depths; (ii) gelation stage, where the mixture polymerizes and transforms into a gel under the influence of the hardener; and (iii) post-gelation (aging) stage, during which the gel undergoes dilution, deformation, and degradation, influencing its long-term efficiency and environmental impact

Silicate grouts typically consist of a sodium silicate solution, water, and a hardener (acid or metal salt) that triggers gelation. The sodium silicate acts as the main binder, water regulates the initial viscosity, and the hardener controls gelation time and gel strength. Depending on formulation, two types of gels can form after injection:

- *Soft gel* A softer, more flexible gel that primarily serves to reduce permeability by blocking water flow through soil pores (Fig. 1a).
- *Hard gel* A more rigid gel that increases soil strength by binding soil particles and improving load-bearing capacity (Fig. 1b).

Once injected, the grout permeates the pore space and gradually solidifies, stabilizing the soil and reducing permeability (Littlejohn et al. 1997; Owusu 1982). This process involves four interacting phases: gel, water, air, and soil across distinct spatial scales: (i) micro-scale: inside the gel, containing polymeric chains, various ions, and interstitial water, (ii) meso- or pore scale: contains all four phases independently, forming interfaces: gel-water, gel-soil, gel-air, air–water, and water-soil, and (iii) macro- or Darcy scale: studies the grouting process in a continuum, not resolving the locations of the four phases. The three scales form an intricately coupled system interconnected through groundwater flow and the migration of grouting fluids (water and dissolved components) within the void spaces, not occupied by solid constituents, such as sand particles or gel matrices. These void spaces are characterized by porosity, which varies across scales. At the micro- to pore-scale, porosity is the volume fraction of voids within the gel matrix, through which interstitial water moves (Huyghe and Janssen 1997; Yu et al. 2018). At the pore-scale to macro-scale transition, porosity describes the volume of water-filled voids between soil grains and gel aggregates (Sweijen et al. 2017). This coupled system is often conceptualized as a dual-porosity system (Murad and Cushman 1997; de Vries et al. 2017), similar to swelling clays (Bennethum and Cushman 1996) or to the swelling behavior observed in granular gels (Huyghe and Janssen 1997), in which distinct pore domains coexist and influence fluid flow and transport processes.

Grouting also proceeds through three temporal stages: (i) injection stage: the grout mixture exists in a liquid state, typically comprising water with dissolved components such as sodium silicate, (ii) gelation stage: the liquid grout undergoes a chemical reaction, transforming into a gel and subsequently forming a solid phase, and (iii) aging stage: the gelled grout remains in place, fulfilling its intended function, such as permeability reduction or soil stabilization (Fig. 1).

Although sodium silicate has been extensively studied in surface construction and materials science (Giannaros et al. 2016), its behavior in underground environments and long-term interactions with groundwater remain poorly understood (Dekker et al. 2020; Krizek and Spino 2000; Spacagna et al. 2017; Tognonvi et al. 2011). These knowledge gaps become critical on larger, deeper construction sites, with extended service lifetimes, and in environmentally sensitive conditions. In this review, we analyze how silicate grout interacts with soils and groundwater across multiple scales and stages, highlight the processes that control efficiency and durability, and outline future research needs to optimize its application in geotechnical engineering.

## Processes and Stages of Grouting

### Injection Stage

#### Injection Methods and Field Implementation

Grouting in geotechnical engineering is commonly performed using one of three main techniques: compaction grouting (Shrivastava and Zen 2018; Ye et al. 2020), fracture grouting (Zhu et al. 2024), and permeation grouting (Park and Oh 2018), each characterized by different pressure regimes and soil responses (Minto et al. 2016; Zou et al. 2018). Compaction grouting uses a highly viscous, low-mobility mixture that densifies soils without entering their pores, enhancing strength through displacement (Shrivastava and Zen 2018). Fracture grouting propagates fractures under high injection pressures to fill and stabilize low-permeability formations (Minto et al. 2016; Zou et al. 2018). Permeation grouting, the focus of this review, introduces a low-viscosity fluid that permeates the pore space under controlled low pressure without disturbing the existing soil structure or fracturing it (van der Stoel 2001). The performance of permeation grouting depends on soil stability, grain-size distribution, and hydraulic characteristics, as well as operational parameters such as injection pressure, flow rate, and grout viscosity (van der Stoel 2001).

**Fig. 2:**
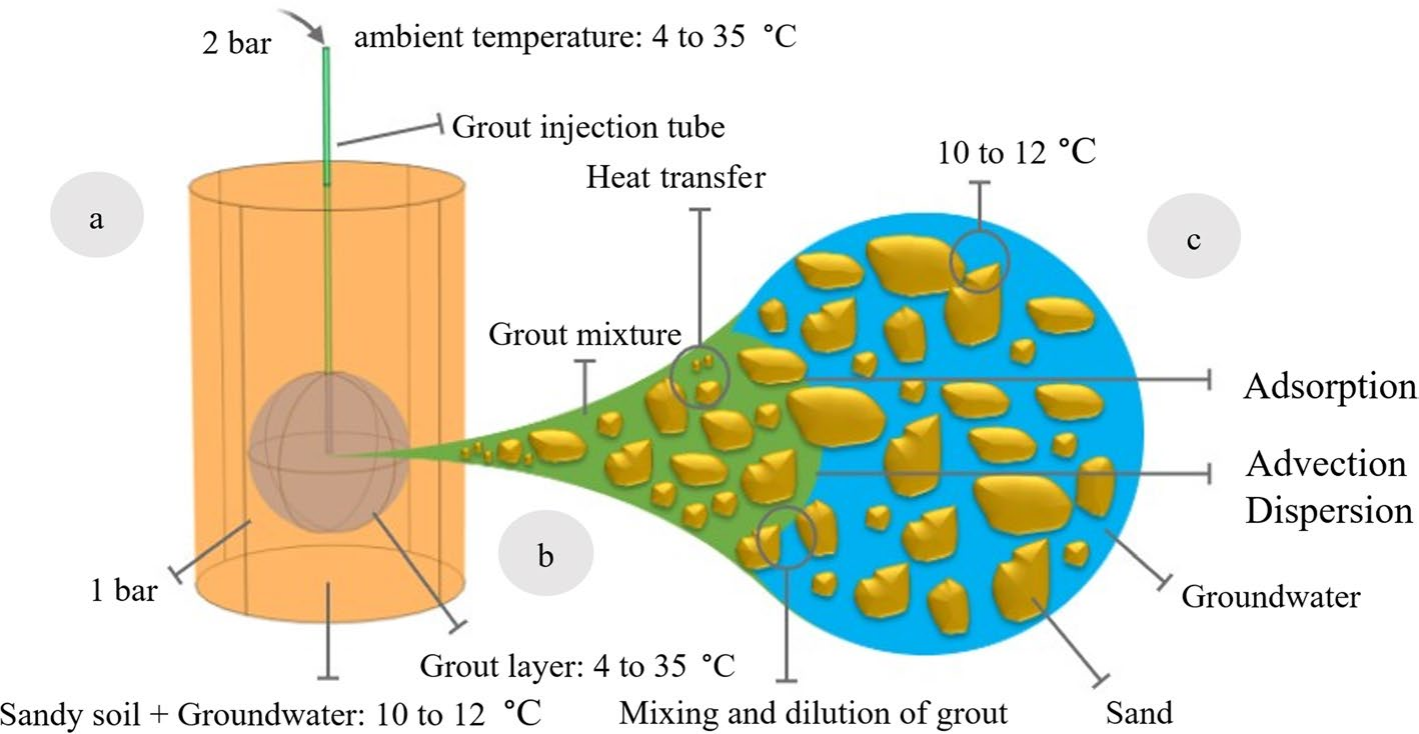
Injection stage: Panel **a** depicts the spherical injection layer surrounding a tube. At a depth of 10 m, where the pressure is approximately 1 bar, an injection pressure of 2 bars is required (van der Stoel 2001). Panel **b** shows the grout front advancing into the sand pores, displacing groundwater, and illustrates the mechanisms involved in the transport of grout components at the pore scale. Panel **c** illustrates the two-phase system of groundwater and sand prior to injection

Figure 2a shows the injection procedure of the silicate grout into the sandy soils. Silicate grouting is typically introduced into soils through a singular point source, often at the tip of a tube or a TAM (tube a manchette or “sleeve pipe”) installed at a specific depth (van der Stoel 2001). The duration of the injection, typically several minutes to hours, depends on the gelation time, injection rate, and the intended treatment volume (Littlejohn et al. 1997; Mollamahmutoğlu and Littlejohn 1995). In uniform sandy soils, the grouted volume assumes a spherical shape, with an effective treatment radius of approximately 0.5 m from the injection point. This radius represents the distance over which the grout penetrates the porous medium before gelation significantly increases viscosity, thereby halting further movement. In practice, grouting is performed by employing multiple injections, arranged in overlapping patterns to create continuous low-permeability layers or reinforced walls (Fig. 1). However, field observations show that injected geometries rarely remain perfectly spherical; anisotropy, layering, and natural fractures in the soil produce irregular shapes and incomplete overlaps (Krizek and Spino 2000). Laboratory studies have extensively examined silicate permeation grouting under controlled conditions (Merrill and Spencer 1950; Panias et al. 2007; Tognonvi et al. 2011), yet field-scale transport and mixing processes remain poorly constrained, limiting predictive design and optimization.

#### Transport Phenomena During Injection

During the injection stage, the system consists of two phases, liquid and solid, since gelation has not yet occurred (Fig. 2c). The injected silicate solution (which is water based) coexists with groundwater within the pore space and behaves as a miscible fluid. Its movement is governed by transport through advection and hydrodynamic dispersion (Fig. 2b). These mechanisms control the spatial distribution of grout components and, therefore, the extent of gel formation and permeability reduction. Dispersion arises from velocity variations at the pore scale and heterogeneity at the macro scale (Bijeljic et al. 2004; Raoof et al. 2010). The transport of the silicate in solution can be described at the macroscale by the advection–dispersion equation for spherical transport (Eq. (1)):

1 $$\frac{{\partial C\left( {r,t} \right)}}{\partial t} = - v\left( r \right)\frac{{\partial C\left( {r,t} \right)}}{\partial r} + \alpha v\left( r \right)\frac{{\partial^{2} C\left( {r,t} \right)}}{{\partial r^{2} }}$$

where *C(r,t)* is the concentration of grout components at radial distance r from the injection point and time *t*, *v(r)* is the flow velocity, *α* is the dispersivity, Assuming a constant dispersivity (*α*) and negligible molecular diffusion, the concentration profile *C(r)* at a particular observation time *t*_*obs*_ can be approximated by the analytical expression presented in Eq. (2) (Gelhar and Collins 1971):

2 $$\frac{{C\left( {r,t_{{{\mathrm{obs}}}} } \right)}}{{C_{0} }} = \frac{1}{2}erfc\left( {\frac{{r^{3} - r_{{\mathrm{c}}}^{3} }}{{6\sqrt {{\raise0.7ex\hbox{${\alpha r_{{\mathrm{c}}}^{5} }$} \!\mathord{\left/ {\vphantom {{\alpha r_{{\mathrm{c}}}^{5} } 5}}\right.\kern-0pt} \!\lower0.7ex\hbox{$5$}}} }}} \right)$$

where *C*₀ is the initial concentration at the injection point, *r*_c_ is the characteristic plume travel distance which is 0.5 m for grouting application, and *erfc* is the complementary error function.

In the subsurface, hydrodynamic dispersion and groundwater flow primarily control how sodium silicate grout dilutes and mixes after injection. For soft gels, whose density is only slightly higher than water, gravity-driven spreading is negligible. Under these conditions, dispersion behaves approximately linearly with concentration gradients, as shown in general miscible-displacement studies where density contrasts are small (Majid Hassanizadeh and Leijnse 1995; Schotting et al. 1999). Mixing is therefore governed mainly by advection and pore-scale velocity fluctuations.

Hard gel solutions typically exhibit substantially higher density and viscosity, which suppresses interfacial velocity fluctuations and reduces apparent dispersivity at the mixing front based on the general miscible-displacement studies (Habermann 1960; Kempers 1991). At these higher density contrasts, buoyancy can become significant. General porous-media studies demonstrate that density differences of this magnitude produce nonlinear dispersion, gravitational fingering, and downward-biased spreading (Majid Hassanizadeh and Leijnse 1995; Schotting et al. 1999). Similar behavior has been documented for dense cement-based grouts: Fu et al. (2019) (Fu et al. 2019) showed that grout self-weight can alter the vertical shape of the permeation bulb and enhance downward migration. Although analogous field observations exist for hard sodium-silicate gels, systematic quantification of gravity-driven spreading in silicate systems has not yet been performed. Future research should therefore investigate the extent of density effects in hard-gel formulations and incorporate these processes into predictive injection models. During grouting applications, the grout experiences varying temperature conditions. Storing the grout in a temperature controlled chamber before injection is generally inefficient and costly, so it is usually injected at ambient temperature; ranging from 4 to 35 °C (Fig. 2a). When injected into the ground, it encounters groundwater temperatures typically around 10–12 °C (Bloemendal and Hartog 2018; Visser et al. 2020). This creates a temperature gradient between the grout and the underground environment.

#### Viscosity Effects During Injection

The viscosity of a silicate grout is a key parameter controlling injectability, penetration distance, and required injection pressure during grouting. An optimal viscosity allows the grout to permeate the pore network without inducing soil fracturing, while maintaining stability against excessive dilution. Typical initial viscosities of sodium-silicate grouts range between 2 and 30 mPa s, depending mainly on silicate composition and total solids content, and remain nearly constant until gelation begins, a desirable characteristic for predictable field performance (Mollamahmutoğlu and Avci 2016). A stable, low viscosity enables uniform infiltration without the need for high injection pressures that could disturb the surrounding formation. Viscosity depends primarily on the SiO_2_/Na_2_O ratio, total solids content, temperature, time, and, to a lesser extent, hardener content. At constant solids content, viscosity increases with a higher SiO_2_/Na_2_O ratio, reflecting greater polymerization of silicate species (Littlejohn et al. 1997). Similarly, increasing the total solids content thickens the solution and raises viscosity, whereas higher temperatures reduce viscosity almost linearly (Littlejohn et al. 1997; Mollamahmutoğlu and Avci 2016). Throughout most of the injection period, viscosity remains steady, rising sharply only near the onset of gelation, which marks the transition from a liquid to a solid state (Mollamahmutoğlu and Avci 2016). A higher hardener concentration slightly increases the initial viscosity but primarily affects subsequent gelation kinetics rather than early-stage flow behavior. Because viscosity directly governs flow resistance and grout-front advancement, accurate rheological characterization is essential for predictive modelling of grout propagation.

#### Modelling and Prediction of Grout Propagation

Predicting grout propagation requires coupling hydraulic, rheological, and chemical processes with operational parameters such as injection pressure, flow rate, and soil permeability. Modelling enables assessment of the spatial and temporal evolution of grout flow and solidification, supporting design optimization and performance prediction. Laboratory experiments combined with numerical simulations have provided key insights. Bolisetti et al. (2009) (Bolisetti et al. 2009) demonstrated that viscosity increase during colloidal-silica gelation alters flow patterns and limits penetration in fine sands, while Lin and Gallagher (2006) observed similar effects in larger sand columns. Although these tests illuminate the role of rheology, they are restricted to specific soils and grouts; site-specific replication is costly and time-consuming. Numerical models therefore offer an efficient predictive tool, provided that essential geo-physico-chemical parameters are available.

Numerous studies (Bouchelaghem 2009; Gustafson et al. 2013; van Lopik et al. 2017; Maghous et al. 2007; Rodríguez De Castro et al. 2019; Yoon and El Mohtar 2015) have delved into modeling the permeation grouting of rocks and sands using cement slurries, considering slurries as Newtonian fluid, Bingham fluid, and power-law fluid. These studies focus on estimating the maximum permeation distance and macroscopic models for the permeation of cement grout. Bouchelaghem et al. (2001) developed an advection–dispersion–filtration model for suspension grouts in deformable porous media, though it requires numerous constitutive parameters. Celik (2019) simplified flow as Darcy’s law scaled by viscosity ratios, neglecting capillarity, whereas Fu et al. (2019) incorporated gravity effects for various rheology models. Coskun and Tokdemir (2020) introduced a miscible-flow model for sodium-silicate grouting, assuming incompressibility and negligible gravity or capillary effects in fully saturated rigid soils. Their predictions matched laboratory results by Honma (1984), although pressure distributions were not fully captured due to simplified Newtonian assumptions. Recent studies include time-dependent viscosity to represent gelation during injection, providing improved agreement with observed behavior (Boschi et al. 2024).

#### Optimization of Injection Stage

Grouting represents a multiphysics process involving coupled momentum, mass, and energy transfer. While the penetration length of grout is often used as a measure of efficiency, understanding the spatial distribution of grout components and the associated temperature fields during injection is equally critical. Coupled transport modelling provides a framework to analyze these interactions, enabling optimization of injection parameters and enhancement of overall performance. Efficiency can ultimately be defined through the spatial and temporal distribution of grout components, allowing the identification of threshold concentrations for effective gel formation that depend on grout composition and hardener chemistry.

Future research should focus on developing constitutive relationships for key parameters including density, viscosity, dispersion coefficient, and thermal properties as functions of temperature and concentration. Such data are essential for predictive modelling of silicate-grout behavior. Advanced modelling tools could further integrate optimization algorithms to determine the optimal configuration of injection points, injected volume per point, and flow rate, thereby preventing excessive material use while ensuring the formation of a continuous and effective grouted layer.

### Gel Formation Stage

Following the injection phase, during which the grout and groundwater coexist as miscible fluids within the pore space, the system transitions into the gelation stage, where chemical reactions dominate. At this stage, the dissolved silicate species begin to polymerize and form a three-dimensional silica network, converting the fluid grout into a gel. Gelation occurs primarily at the micro- and pore scales, where polymeric silicate chains nucleate and grow, often adhering to soil grain surfaces or interacting with pore water flow (Dimas et al. 2009). These coupled chemical and physical processes control the initial gel saturation, microstructure, and mechanical integrity of the treated soil. The gelation process typically occurs over timescales of minutes to several hours (Mollamahmutoğlu and Littlejohn 1995; Wilhelm and Kind 2014). Single-shot silicate-based grouts typically consist of three primary components: sodium silicate, water, and a hardener. Once injected, the hardener undergoes hydrolysis, gradually neutralizing the alkaline silicate solution. When the degree of neutralization reaches a critical level, polymerization accelerates and the mixture solidifies into a white silica gel. Initially injected as a low-viscosity fluid, the grout permeates the soil matrix before gelation increases viscosity and immobilizes it in place. The resulting gel forms a coherent network that reduces permeability and enhances soil strength (Littlejohn et al. 1997).

The proportion of each component in the mixture determines the type of gel formed: soft gels and hard gels (Aurand et al. 1981). Soft gels consist predominantly of water (80–85% by volume), with sodium silicate solution comprising 10–20% and the hardener constituting 1–5%. In contrast, hard gels contain a higher proportion of sodium silicate solution (50–60%) and hardener (6–10%), resulting in lower water content (30–40%). Between these two extremes lie various intermediate gel types (Littlejohn et al. 1997; Tognonvi et al. 2011).

#### Structure of Sodium Silicates

**Fig. 3:**
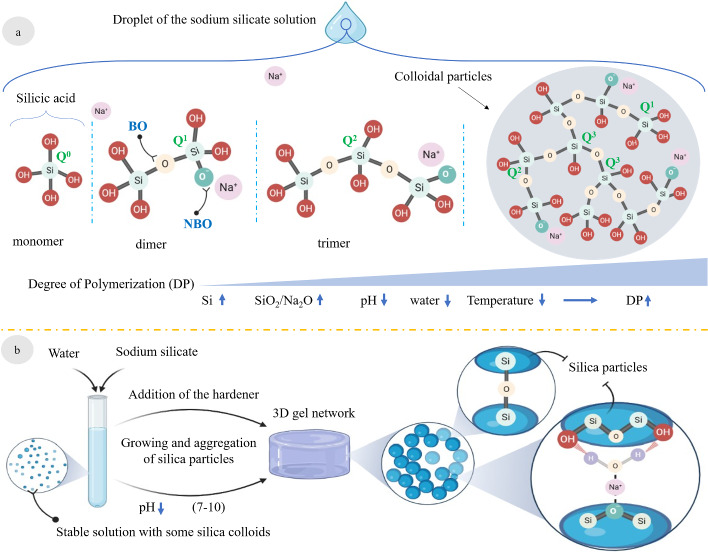
Illustration of silicate speciation and gelation in sodium silicate solutions: **a** transition from monomers (Q⁰) and low-order oligomers (Q^1^–Q^2^) to higher-order polymers and colloidal particles under varying conditions such as pH, concentration, and temperature. Key processes include acid–base equilibria, polymerization–depolymerization reactions, and the formation of Na⁺-associated silicate species (Matinfar and Nychka 2023). Bridging oxygens (BO) connecting neighboring silicate tetrahedra and non-bridging oxygens (NBO) stabilized by Na⁺ ions are shown. Silicate structural units are labeled using the Q^n^ notation, where Q denotes a four-coordinated Si atom and n represents the number of neighboring Si atoms linked through BO atoms (Matinfar and Nychka 2025). **b** Aggregation of silicate particles into larger structures and eventual formation of a three-dimensional gel network. Following Iler’s theory (Ralph 1979), polymerization proceeds through three stages: (i) condensation of silicic acid monomers into oligomers and colloidal particles, (ii) growth of these particles, and (iii) aggregation into branched chains and networks that span the liquid medium and form a gel. Bonding between silica colloids can occur through siloxane linkages (Si–O–Si), hydrogen bonding, and electrostatic interactions (right hand side in b)

Sodium silicates are combinations of sodium oxide, an alkali metal oxide, and silica, typically containing water. Its general formula is represented as: x SiO_2_:Na_2_O where x is the molar ratio (moles of SiO_2_/moles of Na_2_O). The ratio of a silicate solution can also be expressed on a weight basis. For instance, a silicate solution containing 36% SiO_2_ and 18% Na_2_O is described to have a weight ratio of 2:1. Due to the similar molecular weights of Na_2_O and SiO_2_, the disparity between weight and molar ratios for sodium silicate solutions is minimal and often neglected (Liu and Ott 2020; Matinfar and Nychka 2023). For grouting applications, silicates with a molar or weight ratio greater than three are typically preferred to achieve low viscosities, practical gelation times and sufficient gel strength. Figure 3a depicts the chemical structure of sodium silicate and the effect of temperature, pH, and the ratio of SiO_2_: Na_2_O on the degree of polymerization. A higher concentration of Si, a higher SiO_2_: Na_2_O ratio, and lower temperature and pH will result in a higher degree of polymerization (Matinfar and Nychka 2023).

Xu et al. (2022) investigated sodium silicate solutions with varying solid contents and proposed a mechanism for gelation in sandy soils. They observed that higher solid content produced denser gels, enhanced strength, and slower gelation. Gu et al. (2021) examined the influence of solid contents on the physical and mechanical properties of geopolymers derived from fly ash activated by sodium silicate, showing that low molar ratio, high solid content solutions improved cohesion and unconfined compressive strength. Lian et al. (2021) characterized sodium silicate solutions in alkaline systems, reporting that electrical conductivity increased with temperature and silica concentration but decreased with molar ratio, while viscosity increased with silica content and declined linearly with temperature. Experimental results on the permeability of grouted soils further demonstrated that permeability decreased by approximately two orders of magnitude as the silicate content increased from 6 to 8% of soil weight, maintaining a constant silicate-to-hardener ratio. Above ≈ 9.5% silicate content, no further reduction occurred, indicating an optimal grout composition (Elektorowicz et al. 2008). Increasing the calcium-chloride hardener from 2.7 to 5.3% of soil weight produced negligible additional improvement (Elektorowicz et al. 2008). Table 1 summarizes the principal physicochemical properties of sodium silicate solutions and the effects of their variations on grout behavior and the resulting silica gel.

**Table 1:** Physicochemical properties and component of sodium silicate and their impact on the resulting silica gel

SiO_2_	Influences the mechanical properties of the gel, with higher silica levels resulting in stronger gels (Littlejohn et al. 1997)
Na_2_O	Constituent that is neutralized to induce gelation and governs the alkalinity and reaction rate (Matinfar and Nychka 2023)
H_2_O	High water content results in an overly dilute solution, while low water content leads to a highly viscous solution that is hard to pump
%SiO_2_ + %Na_2_O	Solid content of the sodium silicate solution: Governs the density and relative density. A higher relative density results in greater strength and cohesion but slower gelation and diffusion (Gu et al. 2021; Matinfar and Nychka 2023; Xu et al. 2022)
SiO_2_/Na_2_O	Molar ratio: optimum for grouting ≈ 3.0–3.3. Controls alkalinity and polymerization, low modulus promotes fast gelation, whereas high modulus yields stronger and less soluble final gels (Dimas et al. 2009; Xu et al. 2022). Low modulus gels contain more Q^2^ and Q^1^ units resulting in more non-bonding oxygen (NBO) which makes them less stable. High modulus gels contain more Q^3^ and Q^4^ units, resulting in highly crosslinked and stable gel (Dimas et al. 2009)
additives (Al, B, Fe, P, etc.)	Can lower the minimum SiO₂/Na₂O ratio required for stable, durable gels, key for cost reduction and performance optimization (Dimas et al. 2009)
Electrical conductivity	Increases with temperature and silica concentration, but decreases with modulus. At 50 g L^−1^ of SiO_2_ and modulus of 1 at 25 °C is 433 [mS cm^−1^] (Lian et al. 2021)
Viscosity	Rises with silica content and decreases linearly with temperature (Lian et al. 2021). Considering molar ratio of SiO_2_ to Na_2_O at 3.0 to 3.3, Viscosity (20 °C) is 0.075–0.15 [Pa s] (Avci 2017)
Surface tension	Decreases with silica concentration and temperature, confirming that sodium silicate behaves as an oligomeric surfactant. At 50 g.L^−1^ of SiO_2_ and modulus of 1 at 25 °C is 58 [mN m^−1^] (Lian et al. 2021)
Density	Considering molar ratio at 3.0 to 3.3 (20 °C): 1.37–1.39 [gr cm^−3^] (Avci 2017; Wang et al. 2023c)
pH	Considering molar ratio at 3.0 to 3.3 (20 °C): 11.66 [-] (Avci 2017)

#### Hardener

Sodium silicate solutions are alkaline, and neutralization of the alkalinity is needed to form a gel (Avci 2017). The polymerization of the silica gel follows Iler's (Ralph 1979) theory, which occurs in three main stages:

- *formation of primary particles* Silicic acid monomers (Si(OH)_4_) polymerize into oligomers and then into discrete colloidal particles (Eq. (3)). This stage is governed by the pH of the solution, with lower pH favoring condensation over hydrolysis, leading to the formation of less hydrolyzed silicate species (Fig. 3a).
  3 $$2{\mathrm{Si}}({\mathrm{OH}})_{4} + {\text{ H}}^{ + } \Leftrightarrow {\text{ Si}}_{2} { }\left( {{\mathrm{OH}}} \right)_{7} + {\text{ H}}_{2} {\mathrm{O}}$$
- *particle growth* The primary particles grow further, where larger and/or less soluble particles grow at the expense of smaller ones (Lazaro et al. 2013).
- *particle aggregation and gel formation* Under conditions where salts are present or pH is reduced, the particles aggregate into branched chains and networks, forming a three-dimensional gel structure. The aggregation process is influenced by electrostatic and hydrogen-bond interactions and the specific spatial configurations of the silicate species in solution (Fig. 3b) (Matinfar and Nychka 2025).

The diversity of chemical agents used to trigger silicate gelation, including acids, salts, organic hardeners, nanoparticles, and evaporative processes, are summarized in Table 2. These studies collectively illustrate how hardeners influence gelation kinetics, syneresis, dissolution, and the structural evolution of silica networks, and how these effects are characterized using methods such as SEM, XRD, FTIR, NMR, and rheometry. In practice, grout performance cannot be evaluated solely in solution because gelation behavior and mechanical performance depend strongly on the soil environment. Soil mineralogy, surface charge, organic content, and pore structure can accelerate or inhibit gelation, modify syneresis, alter permeability, and determine long-term stability. grout–soil interactions have therefore been studied across a wide variety of sands, loess, carbonate and limestone cores, sandstone, and engineered soils. These findings are consolidated in Table 3 based on the used hardener and soil type, which highlights how different chemical systems behave under realistic geotechnical and hydrogeological conditions. Insights from the studies listed in Tables 2 and 3 are synthesized in the following sections. The full, detailed versions of these tables are provided in the supplementary material (Table S1).

**Table 2:** Overview of hardeners used in silicate gelation and their experimentally observed effects on gelation kinetics, syneresis behavior, dissolution, microstructure, and stability, together with the analytical methods employed in each study (silica gel)

Hardener type	Experimental focus and methods
Boric acid and phosphoric acid	Raman spectroscopy and SEM characterization; comparison of acidic (pH 3–5) vs. basic (pH 9–10.6) gels (Matinfar and Nychka 2025)
	Compression testing and SEM imaging after freeze-drying (Matinfar et al. 2025)
	Gelation kinetics; UV–Vis spectroscopy and Tyndall effect for optical monitoring; SEM microstructure (Matinfar and Nychka 2024)
Silica gel (no hardener)	Dissolution kinetics in LiOH, NaOH, KOH, RbOH, and CsOH solutions; ^29^Si NMR and β-silicomolybdate analysis (Wijnen et al. 1989)
Citric acid + SiO₂ nanoparticles	Gelation time, nanoparticle stability, nucleation effects, and pre-flush performance (25–100 °C) (Hashemi et al. 2023)
Evaporation (drying-controlled hardening)	Effect of silica modulus on Qⁿ structure, dissolution, and gel stability; FTIR, XRD, EDS, SEM (Dimas et al. 2009)
Sulfuric acid (H₂SO₄)	Rheology of gelation (15–35 °C); semi-empirical model for gelation time (Quarch and Kind 2010)
	Standard rheological characterization of silica gelation, thixotropy, and model fitting; SEM (Katoueizadeh et al. 2021)
	Natural and induced syneresis; empirical model; temperature and pressure effects (20–60 °C) (Wilhelm and Kind 2014)
	Influence of pH and sample size on syneresis (Wilhelm and Kind 2015)
Hydrochloric acid (HCl)	Gelation and influence of dissolved salts (Gorrepati et al. 2010)
	Gelation time, syneresis; Cryo-SEM and XRD (Tognonvi et al. 2011)
	Gelation, syneresis, dissolution and stability; pH effects; FTIR (Tognonvi et al. 2019)
	Influence of pH, temperature, salinity, shear rate, and divalent ions on gelation kinetics, gel strength, and syneresis; predictive models (Hamouda and Amiri 2014)
NaCl	Gelation kinetics, strength, and yield pressure; rheometry and DMA (40–60 °C) (Pham and Hatzignatiou 2016)
NaCl, MgCl_2_, CaCl_2_	Laboratory and field study of silicate grout as a sealing in salt formations; gelation time, permeability, and syneresis (Engelhardt and Von Borstel 2014)

**Table 3:** Overview of hardener–soil combinations examined in the literature for silicate grouting, highlighting key experimental findings, mechanical and hydraulic outcomes, microstructural characterization, and long-term durability or environmental considerations (grouted soil)

Hardener—Soil type	Research focus and experimental methods
Phosphoric acid—Medium sand	Gelation time; strength testing; XRD; SEM; sand–gel interaction (Wang et al. 2023c)
Boric acid—River sand	Gelation time; syneresis; strength testing; permeability testing (Mollamahmutoğlu et al. 2017)
Evaporation—River sand	Temperature effects (20–100 °C) on silicate-grouted sand; SEM; strength testing (Xu et al. 2023)
	Influence of silica modulus and relative density on gelation and strength; SEM and NMR of sand–gel interaction (Chen et al. 2023)
Evaporation—Loess	Strength testing; SEM; porosity; XRD; empirical model for UCS (Guo et al. 2024)
Evaporation—Fine sand	Strength testing; SEM; NMR; sand–gel interaction (Xu et al. 2022)
NaCl—Carbonate cores	Gelation time; strength testing; permeability testing (Hatzignatiou et al. 2016)
HCl—Quartz sand columns	Experimental and numerical investigation of bulk gelation; dynamic coreflood experiments (Hatzignatiou et al. 2014)
NaCl, HCl, HNO₃, HCOOH, and urea—Chalk and sandstone cores	Filterability; injectivity; gelation time; strength; shrinkage; dissolution (20–90 °C) (Hatzignatiou and Giske 2018)
Urea—Limestone cores	Bulk gelation and coreflood experiments; gelation time; activation energy; permeability reduction (Nasr-El-Din and Taylor 2005)
Dihydrogen phosphate—River	Gelation time; syneresis; permeability; strength (Mollamahmutoğlu et al. 2021)
Sodium aluminate—Urban aquifer	Two-year field monitoring and laboratory tests; environmental impacts of silica gel; PHREEQE modelling (Eiswirth et al. 1999)
	Groundwater contamination risks associated with soft-gel injection (Eiswirth and HÖtzel 2003)
Formamide + Sodium aluminate—Carbonate sands	Triaxial testing of grouted samples; stress–dilation relationship (Salehzadeh et al. 2012)
Formamide—Silty fine sand	Gelation time; syneresis; strength testing; permeability testing; grouting of fine-grained soil (Avci et al. 2022)
Formamide—River sand	Effects of sand gradation on gelation, syneresis, and permeability (Mollamahmutoğlu and Avci 2020)
Formamide—Quartz sand	Influence of long-term syneresis (720 days aging) on permeability; gelation time; syneresis (Mollamahmutoğlu and Avci 2016)
Formamide—Sandy soil	Laboratory-scale monitoring of chemical permeation grouting using ERT (Pham et al. 2011)
Formamide + Glyoxal + Sodium aluminate—Medium sand	Long-term permeability and chemical durability under exposure to industrial chemicals and real-site wastes (Bodocsi and Bowers 1991)
Glyoxal—Quartz sand	Viscosity; gelation time; syneresis; permeability; influence of sand size (Avci 2017)
Glyoxal + Phosphoric acid—Mikawa sand	ERT delineation of chemically grouted zones; grout–void ratio; grouting effectiveness (Komine 2000)
	Use of electrical resistivity to assess grouting quality and extent (Komine 1997)
Ethyl acetate + Formamide—Maryland sand	Field testing; syneresis; permeability; strength; NaOH-induced dissolution (Krizek and Spino 2000)
Ethyl acetate + Formamide + Calcium chloride—Sand columns	Salinity effects on dissolution and permeability of grouted sand and pure gel (Elektorowicz et al. 2008)
Ethyl acetate + Formamide—Ottawa sand and limestone	Cohesive grout strength and adhesive sand–grout bonding; aging effects; linkage between gel cohesive/adhesive strength and grouted-sand UCS (Ata and Vipulanandan 1998)
	Mechanistic two-phase composite model for chemically grouted sand governed by grout cohesion, sand–grout adhesion, and particle interaction (Vipulanandan and Krizek 1986)
	Dynamic, cyclic, and fatigue behaviour of silicate-grouted sand (Vipulanandan and Ata 2000)
600B (ester)—Leighton Buzzard sand	Viscosity; syneresis; gelation time; groutability; permeation; compressive strength (Mollamahmutoğlu and Littlejohn 1995)
	Temperature effects on creep behavior of grouted sand (Mollamahmutoglu and Littlejohn 1997)
	Influence of silicate content and confinement pressure on strength (Littlejohn and Mollamahmutoglu 1994)
R100 ester—Leighton Buzzard	Viscosity; gelation time; syneresis; compressive strength; permeability reduction; effects of neutralization degree (Littlejohn et al. 1997)
Diacid ester—Silty fine sand	Gelation time; strength testing; SEM; IR; field testing (Cui et al. 2022)
Medium and fine sand	Chemical stability of silicate grout against acids, bases, salts, and organic wastes (May et al. 1986)
Dimethyl ester—Medium sand	Gelation time; syneresis; strength; correlation between gelation time and strength (Gonzalez and Vipulanandan 2007)
Organic hardener—Fontainebleau	Comparison of silicate grout with microfine cement and mineral grouts; effect of sand packing on strength (Delfosse-Ribay et al. 2006)
Organic hardener—Fine to coarse	Influence of particle size, sand surface area, and gel tensile strength on grouted-sand strength (Kaga and Yonekura 1991)
Dibasic ester, ethyl acetate, and formamide—Ottawa sand	Leaching of organics from sodium-silicate grouts; environmental impacts on groundwater (Malone et al. 1995)

#### Gelation Time

Assessing the performance of grouting slurry hinges significantly on its gelation time or setting time, as it usually determines the specific time frame for injection. Qualitative methods report the gelation time based on visual observations of viscosity, thus the gelling time is either the moment that solution stops flowing upon tilting or inversion (Berrier et al. 2010; Tognonvi et al. 2011) or when a significant change in transparency occurs. These methods are simple and inexpensive but rely on the observer’s judgment. Quantitative methods use rheological measurements for higher sensitivity and accuracy. One method defines the gel point as the inflection where static shear viscosity rises sharply (Al-Anazi et al. 2011; Hamouda and Amiri 2014). However, continuous shearing may affect gel formation. Oscillatory rheometry studies viscoelastic behavior by calculating the storage modulus (G') for elastic behavior and the loss modulus (G") for viscous behavior. The gel point is identified when G' exceeds G" (G' = G") (Hatzignatiou et al. 2016; Pham and Hatzignatiou 2016; Quarch and Kind 2010).

Factors affecting sodium silicate gelation (seconds to hours) are listed in Table 4. For practical purposes, the injectability period of a grout spans around 75–80% of the gelation time. For example, if the gelation time is 1 h, injection should be completed within about 45 min (Littlejohn et al. 1997). Grout formulations exhibiting minimal viscosity increase during injection followed by prompt gelation offer the most practical functionality (Littlejohn et al. 1997). Grouts boasting prolonged setting times demonstrate enhanced cementation properties, increased pore filling capacity, and an increased concentration of silica gel (Wang et al. 2023c). Nevertheless, when considering underground injection, opting for longer gelation times proves impractical due to cost efficiency concerns. Longer gelation times necessitate extended waiting periods between injections at adjacent points, thereby impacting operational expenses.

**Table 4:** Parameters affecting the gelation of the silicate grout and resulting gel

Nature of the silicate	Lower modulus generally leads to longer gelation times. Silicates with higher solids concentrations typically yield shorter gelation times, assuming a constant degree of neutralization (Tognonvi et al. 2011)
Silicate concentration in the grout	Holding hardener proportions constant, gelation time increases with higher silicate levels. However, at a consistent order of hardener proportions, higher silicate levels result in shorter gelation times (Hatzignatiou and Giske 2018; Tognonvi et al. 2011)
Hardener	Various commercially available hardeners offer different gelation time characteristics: some are designed for delayed setting in hot climates. As hardener proportion in the grout formulation rises, the degree of neutralization increases, leading to shorter gelation times (Littlejohn et al. 1997)
Temperatures	Numerous studies demonstrate that gelation time decreases exponentially with temperature increase. The Arrhenius-type relation is reported across both acid-activated and salt-activated systems. 10 °C rise results in approximately 2–3 times acceleration of gelation in NaCl-activated systems (Pham and Hatzignatiou 2016). Urea-activated silicate requires T > 70 °C to initiate gelation due to temperature-dependent urea hydrolysis (Nasr-El-Din and Taylor 2005). In acid-initiated systems, gelation is fastest at strongly acidic or neutral–slightly alkaline pH; temperature further accelerates both condensation and particle aggregation (Quarch and Kind 2010). In brines or mineralized groundwater, temperature enhances Ca^2^⁺/Mg^2^⁺-driven gelation (Hamouda and Amiri 2014), promoting denser Ca/Mg–silicate formation at high temperature. Overall, higher temperatures shorten gelation window and promote rapid network formation, which may risk premature setting during injection in warm aquifers or deep formations
Nature of the soil	Soil acidity or groundwater alkalinity tends to respectively shorten or lengthen gelation times (Littlejohn et al. 1997)
pH	Rapid gelation occurs at strongly acidic conditions (pH < 2) and near-neutral to slightly alkaline conditions (pH 2–7). The gelation time increases as the pH moves above 7, reaching a point where no gelation occurs at very high pH levels (above 11) due to the repulsion between charged particles (Brinker 1988; Matinfar and Nychka 2023; Tognonvi et al. 2011). Basic gels form large, loose secondary particles (pores ~ 5 µm); Acidic gels form small, compact ones (< 550 nm). Basic gels dominated by Q^3^ (53–80%), Q^2^ (10–17%), Q⁰ (5–34%); Acidic gels dominated by Q^2^ (62–80%) and Q⁰ (20–38%) (Matinfar and Nychka 2025). In the basic region, gelation time follows an exponential relation with pH; in the acidic region, a third-order polynomial. Gelation kinetics mainly depend on pH and waterglass concentration, not acid type (Matinfar and Nychka 2024). Acidic gels are stronger (higher elastic modulus, G′) than alkaline gels at equal solids content (Quarch and Kind 2010)
Metal ions	Sodium silicate solutions with higher ionic strengths (achieved by adding salts) tend to gel more quickly (Quarch and Kind 2010). Salts accelerate polymerization in order: AlCl₃ > CaCl₂ > MgCl₂ > NaCl > CsCl > no salt (Gorrepati et al. 2010). Divalent cations are more effective in accelerating gelation than monovalent ions (Visser 2018). Presence of Al^3^⁺, Cu^2^⁺, Pb^2^⁺, Nd^3^⁺ delays gelation, forming dense microporous gels with ~ 3.7 nm pores; Na⁺, K⁺, Ca^2^⁺ accelerate gelation, producing mesoporous gels (7–9 nm pores) (Berrier et al. 2010; Hatzignatiou and Giske 2018). Ca^2^⁺ and Mg^2^⁺ ions shortened gel time (Pham and Hatzignatiou 2016) and increase gel strength up to threefold; Mg^2^⁺ slightly less effective (Hamouda and Amiri 2014). Elevated levels of calcium or magnesium (above ~ 200 ppm) may induce premature gelation due to the formation of highly insoluble silicate salts(Ca/Mg–silicate formation) (Nasr-El-Din and Taylor 2005) which can be prevented by Citric acid pre-flush to mask Ca^2^⁺ and Mg^2^⁺ (Hashemi et al. 2023)
Groundwater contamination	Different contaminants influence the gelation time differently. Sodium and ammonium hydroxide, benzene, gasoline, oil, toluene, and trichloroethylene, slowed down or prevented gel formation upon contact. Hydrochloric acid, ammonium chloride, copper sulfate, and phenol, accelerated gel formation (May et al. 1986). Strong bases (NaOH, NH₄OH) inhibited gelation or caused partial dissolution of silicate gels
Soil organic matter	Soil organics can dissolve under alkaline conditions, delaying gelation and weakening the gel network (Elektorowicz et al. 2008), can prevent gelation entirely (Hurley and Thornburn 1971), and induce swelling, softening, and detachment from soil particles, increasing permeability of grouted sand (James H. May et al. 1986)
Dilution	Higher water ratios lead to depolymerization (Q₃/Q₂ → Q₁/Q₀) due to Na⁺ dissociation and Si–O–Si bond hydrolysis (Matinfar and Nychka 2025) and increases gel time and pore size (Matinfar and Nychka 2024)
Pressure	Pressure increase, delays gelation due to enhanced silicate solubility (Pham and Hatzignatiou 2016)

#### Effect of the Grout Dilution on Gel Functionality

**Fig. 4:**
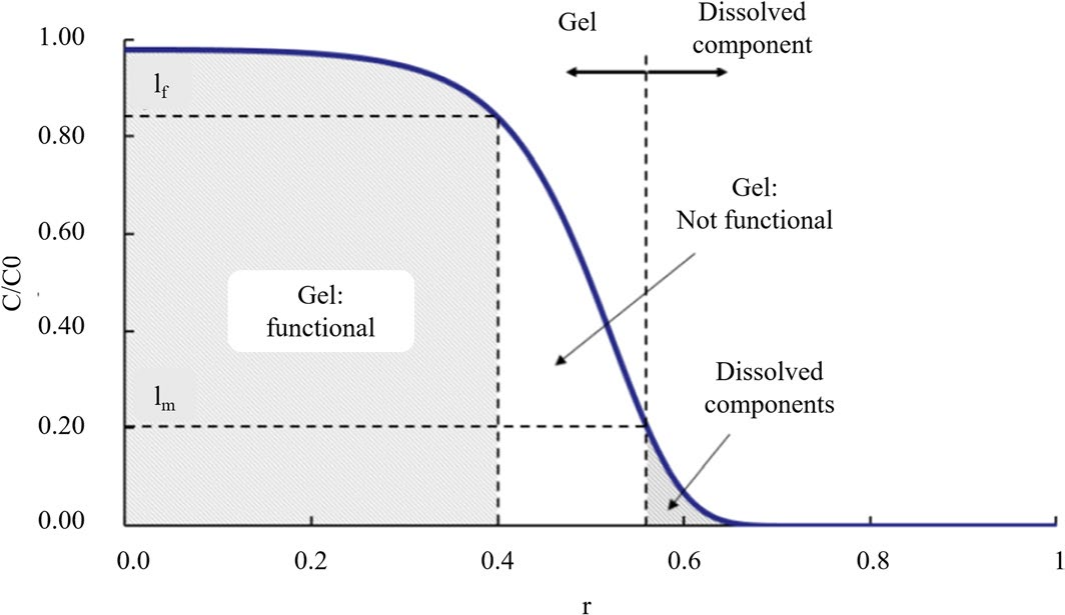
Definition of threshold values of dilution: concentration profile from spherical transport Eq. (2)

During injection, grout mixes with groundwater causing a dilution gradient in the concentrations of grout constituents; including silicate species and hardener. The theoretical concentration profile of grout components from Eq. (2), is illustrated in Fig. 4, showing how concentration decreases with distance from the injection point. The functionality of the resulting gel can be defined using two threshold concentrations: *lf*, where grout becomes a functional gel, and *lm*, where grout is too diluted and remains a miscible fluid. Between thresholds *lf* and *lm*, a gel is formed without its intended properties (Fig. 4). The width of the window of diluted grout concentration (0 < *C(r)* < *C*_*0*_) is a function of the dispersivity (*α*) and thus of the degree of soil heterogeneity.

The threshold value *l*_*f*_ for functional gel primarily depends on practical constraints such as the gelation time. For example, when diluted twice (*C/C*_*0*_ = 0.5), the gelation time for soft gels increases by a factor of 2 to 7 (Tognonvi et al. 2011). For hard gels, the gelation time increases from 30 to 110 min (a factor of 3.7) when the concentration is diluted to 70% of its initial concentration (*C/C*_0_ = 0.7) (Mollamahmutoğlu and Littlejohn 1995). In practice, silicate grouts are designed to set within minutes to an hour after injection. Increasing the gelation time will enhance the risk that silicate grout is pushed away before setting by a neighboring injection that typically is placed within hours after the first injection. We assume the value of *l*_*f*_ to be between 0.5 and 1.0 and thus the radius at which *C(r)/C*_0_ = *l*_*f*_ to be lower than the hydraulic radius at which *C(r)/C*_0_ = 0.5.

The second threshold, *l*_*m*_, below which grout remains miscible, is primarily determined by a physical constraint for grout to form a gel (Tognonvi et al. 2011). Tognonvi et al. (2011) (Tognonvi et al. 2011) reported that the gel did not set at all for values of 0.2 mol/l or lower. This indicates threshold values of *C(r)/C*_0_ < 0.2 for soft gels and *C(r)/C*_0_ < 0.06 for hard gels (Tognonvi et al. 2011). Threshold values depend on several factors, including the hardener type, chemical composition, and temperature. Further experimental research quantifying these thresholds for different grout formulations is needed for a more accurate predictive design and optimization of silicate-grouting applications.

#### Sand-Gel Interaction

The interaction between sodium silicate gel and sand grains is central to the hydraulic and mechanical performance of silicate‐grouted soils. Unlike free-standing gels, which typically undergo severe syneresis, cracking, and dissolution, sand–gel composites exhibit markedly enhanced dimensional stability and strength due to a combination of physical confinement and interfacial chemical bonding. These interactions operate across scales and evolve significantly during curing and aging.

**Fig. 5:**
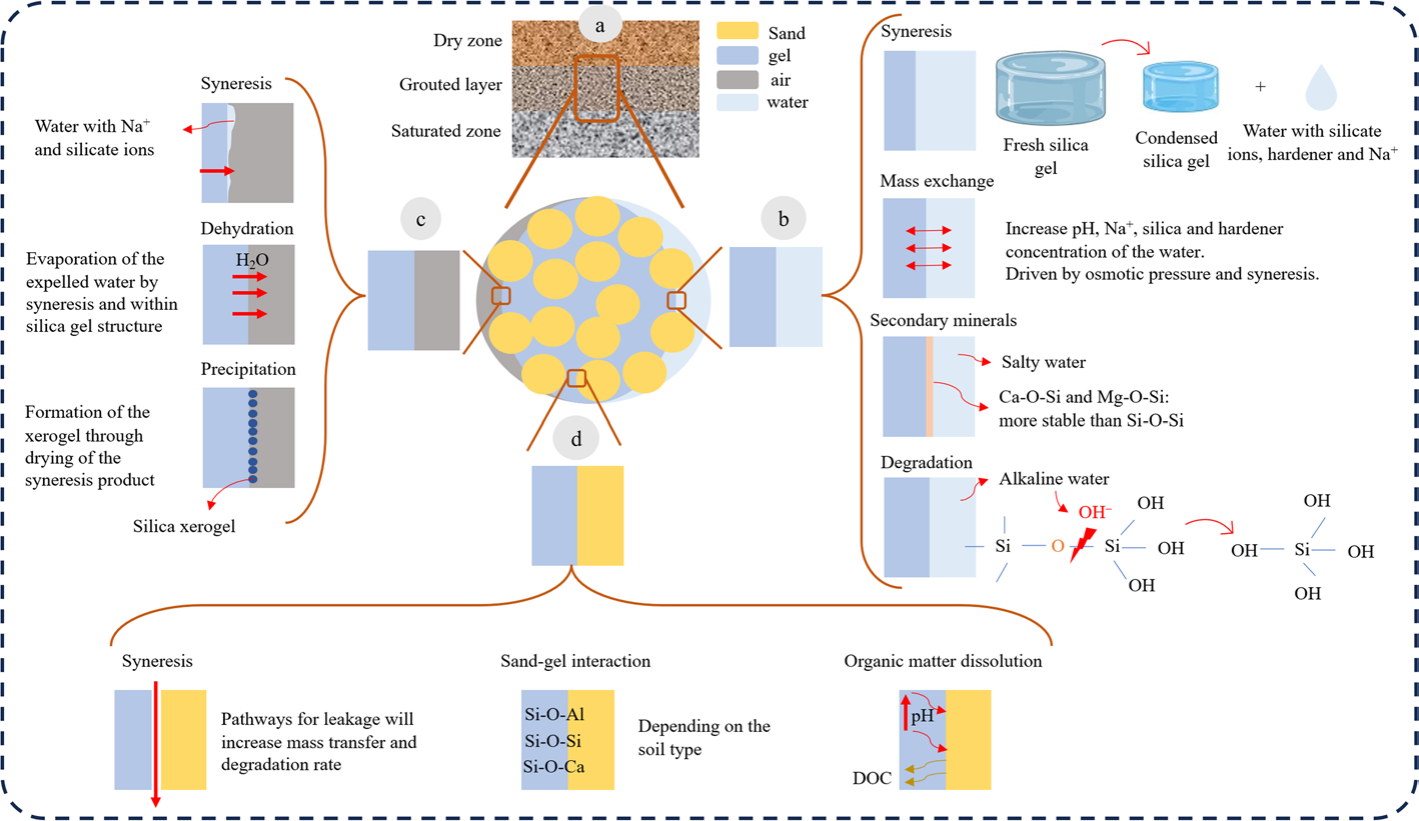
Schematic illustration of the processes occurring during the aging stage: **a** positioning of the grouted layer relative to the saturated and unsaturated zones. Soft-gel applications occur entirely within the saturated zone, whereas hard-gel applications can span both saturated and unsaturated domains. The inset shows the pore-scale configuration of sand, gel, water, and air. **b** Enlarged view of the gel–water interface, highlighting key processes: gel syneresis and degradation, secondary-mineral formation at the interface, and leaching of gel components into groundwater. **c** Enlarged view of the gel–air interface, illustrating gel syneresis, evaporation of syneresis water and pore water within the silica network, and subsequent drying of expelled silicate species leading to the formation of silica xerogels. **d** Enlarged view of the gel–sand interface, showing syneresis-induced detachment of the gel from sand grains and the creation of preferential flow paths, chemical anchoring of the gel to mineral surfaces, and dissolution of organic matter under high-pH conditions

When sodium silicate solution permeates silica-rich sands, hydroxylated quartz surfaces (≡Si–OH) serve as highly reactive sites for rapid adsorption of silicate monomers and oligomers. Under alkaline conditions, Na⁺ attack on quartz and aluminosilicates releases monomeric silicate species, which subsequently condense to form new interfacial Si–O–Si and Si–O–Al bonds (Fig. 5d) (Chen et al. 2023; Xu et al. 2022). This process produces thin but continuous silica films that encapsulate individual grains and bridge adjacent contacts. As polymerization and condensation progress, these coatings densify into mechanically robust three-dimensional networks, imparting partial cementation and improved stiffness even at modest grout contents.

Pore filling accompanies interfacial bonding. SEM analyses consistently show that low-Baume formulations generate compact, thin coatings with fine pore structures, whereas high-Baume systems produce thicker agglomerates, larger pore clusters, and increased brittleness (Chen et al. 2023; Xu et al. 2022). Well-graded (with wide range of particles size) and fine sands promote uniform gel retention and more extensive bonding because of higher specific surface area (Mollamahmutoğlu and Avci 2020). In carbonate-bearing sands, chemical interaction between Ca^2^⁺ and silicate solution forms Ca–silicate hydrates, yielding denser, more crystalline bonds and stronger interparticle bonds in the grouted composite than quartz sands (Ata and Vipulanandan 1998; Guo et al. 2024). Sodium silicate performs well in sandy or low-plasticity soils but is ineffective in montmorillonite-rich clays where Na⁺ dispersion and surface charge interactions inhibit gel formation.

Temperature also influences the sand–gel interface. Elevated temperatures accelerate Na⁺-promoted dissolution of quartz and aluminosilicates, increasing monomer supply and thus strengthening early-stage bonding (Xu et al. 2023). However, high temperatures also accelerate syneresis-driven interfacial debonding at later stages. Thus, temperature enhances initial adhesion but may intensify long-term shrinkage.

Overall, sand–gel interaction involves a coupled sequence of surface-catalyzed polymerization, pore filling, and progressive densification that create durable intergranular bonds and refine the pore structure. These mechanisms underpin the permeability reduction, stiffness increase, and long-term behavior of silicate-grouted sands. However, most evidence for chemical sand–gel interactions comes from studies in which solidification occurs through gel dehydration (drying) rather than through the use of external hardeners. Further research is therefore needed to clarify how bonding and sand–gel interactions develop in silicate grouts that contain hardeners.

#### Optimization of Gelation Stage

The efficiency and performance of silicate grouting depend strongly on controlling the gelation process to achieve the desired balance between injectability, setting time, and final strength. Optimization of the gelation stage involves tuning the chemical composition (silicate modulus, solid content, and hardener type) and environmental conditions (temperature, pH, and groundwater chemistry) to promote uniform and predictable gel formation. For field applications, grouts should maintain low viscosity during injection and rapid polymerization afterward to minimize dilution and displacement. This can be achieved by adjusting the SiO_2_/Na_2_O ratio to approximately 3.0–3.3, selecting hardeners that provide controlled neutralization rates, and tailoring solid content to balance workability and strength. Given that dilution and groundwater composition can modify gelation kinetics, future optimization strategies should integrate coupled chemical–transport modelling and site-specific geochemical characterization to predict gel behavior under in-situ conditions. Experimental data on the effects of organic content, dissolved salts, and contaminants are particularly needed to refine these predictive models. Ultimately, optimizing the gelation stage aims to maximize uniform gel formation and long-term stability while minimizing excess material use and environmental impact. The post-gelation evolution of the grout, including syneresis, and degradation, further governs the longevity of treated soils and is discussed in the following section.

### Aging Stage

In the aging stage (Fig. 5), all four components (gel, water, air, and soil) are in place. At the micro-scale, processes within the gel, such as shrinkage, swelling, and syneresis, impact macro-scale parameters like permeability and soil strength. At the pore scale, mass transfer occurs at the gel-water interface (Fig. 5b), driven by processes such as syneresis, cation exchange, osmotic pressure differences, or physical and chemical degradation of the gel. At the gel–air interface (Fig. 5c), mass transfer occurs through syneresis-driven expulsion of water from the gel and through dehydration of the pore water within the gel. At the macro-scale, transport phenomena such as advection, dispersion, and diffusion of leached gel products are observed (Schnell 2001). Adsorption of dissolved species might occur downstream of the gel. The timescales of the aging stage range from days for syneresis (Littlejohn et al. 1997) to years for continuous syneresis and aging of silicate gels (Dekker et al. 2020; Schnell 2001; Visser 2018). The silicate gel will exchange mass with the ambient groundwater, which is site-specific due to environmental parameters, building designs, and construction plans.

#### Syneresis and Swelling

Once the gel forms, polymerization persists, facilitating ongoing condensation among and within the silicate particles. This sustained condensation prompts the expulsion of liquid from the gel pores. Consequently, the gel network undergoes consolidation and shrinkage, leading to alterations in its volume, porosity, and internal surface area as time progresses (Tognonvi et al. 2019; Wilhelm and Kind 2014). This water loss (syneresis) causes shrinkage and distortion of the gel structure. The degree of syneresis is defined as the weight of water expelled from the gel, expressed as a percentage of the initial weight of the gel sample (Matinfar and Nychka 2023). Factors contributing to the degree of syneresis are listed in Table 5.

**Table 5:** Factors influencing syneresis and stability of silica gel during aging

Silicate ratio (SiO_2_/Na_2_O)	Syneresis decreases with increasing silicate ratio for a given silicate level and degree of neutralization (Littlejohn et al. 1997)
Degree of neutralization	Syneresis diminishes with a higher degree of neutralization (Littlejohn et al. 1997)
SiO_2_ content	Syneresis increases with an elevated level of silicate in the grout formulation (Mollamahmutoğlu and Avci 2016; Tognonvi et al. 2011)
%SiO_2_ + %Na_2_O	Syneresis increases with the rising sodium silicate content up to a critical point. Beyond this point, further increase in sodium silicate content leads to a decrease in syneresis (Avci 2017; Mollamahmutoğlu and Avci 2020, 2016)
Soil average grain size	Larger grain size results in the increase of the syneresis (Littlejohn et al. 1997; Mollamahmutoğlu and Avci 2016)
pH	A higher pH decreases syneresis (Tognonvi et al. 2011), the maximum syneresis occurs between pH 5 and 10 (Hamouda and Amiri 2014), with the lowest rate happening at the isoelectric point (pH ≈ 2) (Matinfar and Nychka 2023)
Size of gel body	Smaller samples consolidate much faster than larger ones due to their lower permeability (Wilhelm and Kind 2015)
Temperature	Syneresis rate increases with rising temperature due to the facilitation of movement of the pore liquid and faster Si–O–Si condensation (Hamouda and Amiri 2014; Wilhelm and Kind 2015, 2014) but does not significantly affect total shrinkage (Wilhelm and Kind 2014). In sand–gel systems, confinement reduces temperature-induced syneresis, but the trend remains observable (Mollamahmutoğlu and Avci 2016). Therefore, higher temperature accelerates aging (from months to days (Wilhelm and Kind 2014)), densification, and shrinkage, which may increase cracking risk. High-pH environments dissolve silica faster at elevated temperature (Wijnen et al. 1989). High temperature favors Ca-silicate formation, improving stability in mineralized groundwater (Engelhardt and Von Borstel 2014)
Metal ions	Metal ion additives elevate the ionic strength which accelerates syneresis (Hamouda and Amiri 2014)
Salinity/brackish groundwater	In saline water, gels may shrink due to dehydration or ion exchange; stability improves when Na⁺ is replaced by Ca^2^⁺ or Mg^2^⁺ forming denser gels (Elektorowicz et al. 2008; Fu 2019; Hamouda and Amiri 2014; Engelhardt and Von Borstel 2014; Visser 2018)
Contaminants (alkalis, acids, solvents)	Strong alkalis (e.g., NaOH) dissolve gel; acids (e.g., HCl) may preserve or densify it; organic solvents can increase permeability or cause softening. Resistance improves with additives such as aluminate or dimethylformamide (Bodocsi and Bowers 1991; May et al. 1986; Krizek and Spino 2000)
Pressure	Higher external pressure yields faster and greater shrinkage. Natural syneresis shows ~ 20% volume shrinkage (ΔV/V₀ ≈ 0.2) at 20–60 °C, while enforced syneresis (Δp = 15.5 bar) reached 87% shrinkage within hours (Wilhelm and Kind 2015; Wilhelm and Kind 2014)

A consistent finding across experimental studies is that syneresis is much less of a problem in sand–gel systems than in neat gels. For pure gels, volume shrinkage between 20 and 50% is common (Avci 2017; Mollamahmutoğlu and Avci 2020, 2016; Wilhelm and Kind 2015, 2014). By contrast, when the same formulations are injected into sand, reported syneresis is typically confined to a few percent and often has only a modest influence on hydraulic properties (Gonzalez and Vipulanandan 2007; Littlejohn et al. 1997; Mollamahmutoğlu and Avci 2016; Mollamahmutoğlu and Littlejohn 1995; Porcino et al. 2012). Littlejohn and Mollamahmutoglu (1994) and Gonzalez and Vipulanandan (2007) showed that syneresis in permeated sands rarely exceeded 5%, compared with 20–36% in the corresponding neat gels. Long-term monitoring of formamide–silicate systems revealed that permeability increased slightly over months to years due to gel shrinkage, but remained 1–4 orders of magnitude lower than in untreated sands (Mollamahmutoğlu and Avci 2016; Mollamahmutoğlu and Littlejohn 1995).

There is broad agreement in the literature that syneresis in sand–gel systems is lower than in pure silica gel (Gonzalez and Vipulanandan 2007; Littlejohn et al. 1997; Mollamahmutoğlu and Avci 2016), although the underlying mechanisms have not been discussed. Several processes may contribute to this reduction: (i) Pore confinement and pore pressure effects restrict volumetric contraction and promote internal water redistribution rather than free drainage, thereby limiting macroscopic shrinkage. (ii) The rigid grain skeleton constrains deformation, causing strain to localize within thin gel films and bridges at grain contacts; microcracking and densification occur at the interface without generating large voids. (iii) Chemical interactions such as hydrogen bonding and silica–silica or silica–aluminosilicate linkages develop between the gel and mineral surfaces, distributing shrinkage locally instead of volumetrically. These mechanisms require further investigation to clarify the origin of reduced syneresis in sand–gel systems. X-ray imaging of aging grouted sand columns could resolve the spatial extent of syneresis under realistic conditions, while Raman microscopy on silica gel in contact with sand could determine whether interfacial chemical bonding contributes directly to the observed reduction in syneresis.

In contrast, swelling represents the uptake of water into the gel network, primarily driven by osmotic pressure arising from ion hydration and concentration gradients between the gel interior and surrounding groundwater. Upon gel formation, the mobility of cations and anions decreases, shortening Si–O–Si bond distances and redistributing hydration water. Strongly hydrated cations such as Na⁺ (a hard Lewis acid) interact weakly with silanol groups but retain large hydration shells. Their association with the gel surface increases the local osmotic pressure, which promotes swelling. In contrast, weakly hydrated cations such as K⁺ (a softer acid) form weaker interactions with the hard-base silanol oxygens and therefore induce minimal swelling. Multivalent hard acids such as Ca^2^⁺ and Mg^2^⁺ interact more strongly with Si–O⁻ groups and can form inner-sphere complexes that compact the silica network. These ions reduce osmotic gradients, stabilize particle contacts, and suppress swelling by promoting a denser gel structure. Experimental studies have shown that adding calcium hydroxide markedly reduces swelling, likely through the formation of a stiffer, calcium-enriched silica gel (Struble and Diamond 1981; Visser 2018). Similar reduction in swelling is observed in saline environments where Ca^2^⁺ and Mg^2^⁺ replace Na⁺ within the gel structure.

Overall, the balance between syneresis and swelling governs the dimensional stability of the gel during aging. Excessive syneresis can cause volume loss, cracking, and increased permeability, whereas uncontrolled swelling may disrupt soil–gel interfaces or lead to micro fracturing. Managing environmental conditions (pH, salinity, cation concentrations), gel composition, and curing conditions is therefore essential for maintaining the mechanical integrity and hydraulic performance of silicate-grouted soils over time.

#### Degradation of the Silica Gel

Once injected into the subsurface, silica gel undergoes progressive degradation upon interaction with groundwater (Fig. 5b), releasing soluble silica species and associated ions that are transported by advection and dispersion. This degradation reflects coupled microscale depolymerization of the Si–O–Si network and continuum-scale advective leaching of dissolution products. The long-term stability of silicate grouts is strongly controlled by groundwater chemistry, hardener type, gel modulus, and local hydraulic gradients.

Sodium silicate gels degrade by hydrolysis of Si–O–Si bonds, particularly under highly alkaline conditions. Rapid deterioration has been observed in NaOH- and KOH-rich environments, where complete dissolution may occur within hours due to hydroxyl-induced cleavage of bridging oxygens (Hamouda and Amiri 2014; Hatzignatiou and Giske 2018; May et al. 1986). This follows the catalytic depolymerization pathway described by Wijnen et al., (Wijnen et al. 1989), in which OH⁻ enters the Si coordination sphere, forming hypervalent intermediates, weakening Si–O–Si linkages, and yielding monomeric Si(OH)₄ that subsequently oligomerizes into dimers, trimers, and Q^3^-rich species. Dissolution rates increase in the order (LiOH ≈ CsOH) < (RbOH ≈ NaOH) < KOH, reflecting cation hydration and its influence on silicate speciation (Wijnen et al. 1989).

Recent work has revealed that dissolution is often coupled to dissolution–precipitation cycles rather than simple hydrolysis. Tognonvi et al. (2019) (Tognonvi et al. 2019)showed that partially neutralized sodium-silicate gels separate into Na-rich soluble phases (e.g., NaSi_1.87_O_4.24_) and silica-rich, highly crosslinked insoluble phases (e.g., NaSi_12.66_O_25.82_). During dissolution in water, the Na-rich phase leaches readily, releasing nanometric silicate oligomers (0.6–1.2 nm) into solution, whereas the condensed silica-rich phase persists as a low-solubility residue. In gels dominated by physical aggregation (reversible gels), dissolution is complete: the network disintegrates into small colloidal silicates. In irreversible, crosslinked gels, only partial dissolution occurs, and ripening leads to progressive densification of the remaining silica skeleton. These mechanisms explain field observations of early-stage silica mobilization followed by long-term persistence of a more inert residue (Tognonvi et al. 2019).

Silicate gels undergo ion exchange when exposed to saline or brackish water. Fu (2019) (Fu 2019) showed that substitution of Na⁺ by Ca^2^⁺ converts sodium silicate gel into Ca-silicate networks, which are denser and chemically more stable. Hamouda & Amiri (2014) observed that Mg^2^⁺ and Ca^2^⁺ in saline water cause rapid precipitation of Mg- and Ca-silicates at the water–gel interface, a condensation reaction that consumes silicate species and produces a more mineralized interface layer. The strength of this reaction depends on the growth of the gel–water interface, making coarse, permeable soils more susceptible than fine sands with limited interfacial area (Aurand et al. 1981; Fu 2019; Mollamahmutoglu and Littlejohn 1997). In strongly mineralized brines, Engelhardt and Von Borstel (2014) found that sodium silicate reacts rapidly with rock salt, forming halite and amorphous silicates. In MgCl₂-rich brines, the system forms stable magnesium-oxychlorides, low-solubility minerals that generate exceptionally impermeable seals. These reactions raise pH (from ~ 7.2 to ~ 11) and release Na⁺ during Mg^2^⁺ substitution but ultimately yield chemically robust, long-lasting gel–mineral composites.

Contaminant-induced degradation depends strongly on concentration. High-strength NaOH, organic solvents, and some organic derivatives increase permeability through gel shrinkage and softening (May et al. 1986). Bodocsi and Bowers (1991) conducted tests with lower contaminant concentrations using hard gels with an aluminate additive to evaluate the long-term resistance of silicate gels and other grout types against contaminated groundwater. They examined various substances, including sodium hydroxide, cupric sulfate, ethylene glycol, xylene, acetone, aniline, methanol, and hydrochloric acid, showing that silicate grouts exhibit reasonable resistance against chemicals at low concentrations (Bodocsi and Bowers 1991).

Flowing groundwater enhances degradation by transporting dissolution products away, maintaining concentration gradients, and mechanically eroding weakened gel surfaces. Numerical modeling by Dekker et al. (2020) (Dekker et al. 2020) suggests that the hydraulic conductivity of a soft-gel grouting layer may increase from 10^−^⁷ to 10^−4^ m s^−1^ over several years due to dissolution and advection.

Based on field and lab studies, degradation occurs over: (1) days–weeks: early leaching of Na⁺, dissolved organic carbon (DOC), and pH spikes; strong syneresis; clogging of wells due to organic matter dissolution, (2) months: stabilization of groundwater chemistry; onset of permeability increase due to shrinkage, (3) years: gradual dissolution of weak gels, especially soft gels exposed to flowing groundwater or alkaline conditions; partial mineralization in saline settings, (4) decades (in salt formations): gels transform into stable Ca/Mg–silicate phases providing long-term sealing.

This aspect of silicate grouting has received the least attention in the literature, yet it represents one of the most critical stages of the process. To achieve durable and predictable grouting performance, future research should develop a comprehensive framework for silica-gel degradation kinetics that accounts for hardener type, groundwater chemistry, temperature, and confinement by the sand skeleton. Such a framework must be coupled with transport and geochemical modelling tools to predict the extent of dissolution, secondary mineral formation, and the resulting impacts on subsurface hydraulic and mechanical behavior.

## Environmental Impact of Silica Gel

Silicate grouting is often regarded as one of the least toxic chemical grouting methods, yet its interaction with groundwater and soil can lead to several short- and long-term environmental impacts. These impacts arise from three primary sources: (i) incomplete gelation and injection inefficiencies, (ii) leaching of components from the solidified gel through syneresis and degradation, and (iii) engineering or execution errors that allow grout or residuals to enter the groundwater system. Because silica gel constituents occur at concentrations on the order of grams per liter, while groundwater quality standards are typically expressed in milligrams per liter, even small deviations in grouting performance can produce measurable changes in aquifer chemistry.

Immediately after injection, silicate grout can significantly alter groundwater composition. Field monitoring in German aquifers (Eiswirth et al. 1999; Eiswirth and HÖtzel 2003) consistently showed increases in pH to 10–11 and elevated concentrations of Na⁺, dissolved silica, and DOC within 5–10 m downstream of grout curtains. Similar observations were reported by Schnell (Schnell 2001) and in hydrochemical modelling by Dekker et al. (2020), who predicted increased Na⁺, SiO_2_, DOC, and alkalinity in the plume emerging from degrading soft gels. These perturbations typically attenuate within 12–24 months due to dispersion, sorption, carbonate or aluminosilicate precipitation, and dilution. High pH also enhances the dissolution of soil organic matter. Elektorowicz et al. (2008) found that humic substances were mobilized from sandy soils treated with silicate gels, and a field case from The Hague (Luger et al. 2022) documented clogging of dewatering wells due to dissolved organic matter released by alkaline soft gels.

Organic hardeners (ethyl acetate, formamide, dibasic esters) release substantial quantities of organics: more than 70% of initial total organic carbon leaches within the first hours (Malone et al. 1995). Ester-based hardeners undergo alkaline saponification, in which the ester reacts with hydroxide to produce a sodium carboxylate and the corresponding alcohol. Amide-based hardeners, such as formamide, undergo alkaline hydrolysis, producing a sodium carboxylate and ammonia. These products are mobile and biodegradable, causing temporary oxygen depletion and pH shifts (Aurand et al. 1981), leading many European countries to phase out organic hardeners in the late 1990s. Inorganically hardened grouts (e.g., aluminate, phosphate, CaCl_2_) leach only minor concentrations of dissolved Al, Ca, or phosphate, generally < 10 mg/L, and effects are spatially limited (Eiswirth et al. 1999; Elektorowicz et al. 2008).

The available studies on silicate grout impacts (Aurand et al. 1981; Eiswirth et al. 1999; Elektorowicz et al. 2008) focus exclusively on hydrochemical changes such as pH, DOC, sodium, silica, and metal concentrations, without providing any ecotoxicological data on aquatic or soil organisms. Thus, despite detailed hydrochemical analyses, the biological toxicity of silicate-grout leachates remains essentially untested. Although generic ecotoxicology data indicate low inherent toxicity for many individual degradation products, a comprehensive assessment specific to grouting is still required. Such work should examine the full leachate mixture and its variation with different hardener chemistries.

Across the literature, four robust conclusions emerge.

- Environmental impacts are localized and temporary. Changes in pH, DOC, Na⁺, and SiO_2_ typically remain within ~ 10 m of injection zones and return to baseline conditions within 1–2 years (Eiswirth et al. 1999; Eiswirth and HÖtzel 2003; Schnell 2001).
- Organic hardeners pose the highest environmental risk. They generate large quantities of mobile, biodegradable by-products that consume O_2_, alter redox conditions, and may introduce ammonia, carboxylates, or alcohols into aquifers (Aurand et al. 1981; Malone et al. 1995).
- Inorganic hardeners and Ca/Mg-rich groundwater enhance long-term stability. Precipitation of Ca/Mg-silicates immobilizes Na⁺ and SiO_2_, reducing leaching and erosion (Elektorowicz et al. 2008; Engelhardt 2014).
- Environmental risk is dominated by grout design and execution. pH, modulus, hardener type, injection pressure, and groundwater flow control the extent and duration of impacts. Poorly gelled mixtures (low gel index) produce the highest contamination because unreacted sodium silicate is readily transported by groundwater.

Research shows that the environmental impact of silica gels is generally localized and can be effectively controlled through improved grout design and execution. However, new challenges have emerged with modern applications. These include clogging of nearby geothermal wells caused by alkali-mobilized organics or silica precipitates, and the discharge of contaminated pit water resulting from leakage through improperly formed grout layers. These issues highlight the need for future research to understand and mitigate the interactions between silicate grouting, subsurface energy systems, and construction dewatering practices. Based on these findings, environmentally sensitive sites should preferentially use inorganic hardeners (e.g., CaCl_2_, MgCl_2_, NaAlO_2_, phosphates), while organic hardeners should be avoided near drinking-water abstraction zones. Injection volumes should be minimized, and over-pressurization avoided, to prevent unwanted migration pathways. Hydrochemical monitoring over a 1–2 year period, focusing on pH, DOC, Na⁺, and SiO_2_, is recommended.

## Monitoring of Silicate-Grouted Sand Using ERT

Monitoring the distribution and continuity of silicate grout within porous media is essential for evaluating the effectiveness of permeation grouting, detecting defects, and understanding the evolution of grout layers during curing and aging. Electrical resistivity tomography (ERT) (Falzone et al. 2019) has emerged as one of the most effective non-destructive techniques for this purpose, owing to the large resistivity contrast between sodium silicate gel, pore water, and soil minerals. Pure silicate gels typically exhibit resistivities in the range of 0.3–1 Ω m, whereas saturated sands commonly range from 20 to 500 Ω m, depending on pore water conductivity (Komine 2000, 1997; Pham et al. 2011). This sharp resistivity contrast enables high-resolution imaging of grout migration paths, gel continuity, and zones of incomplete penetration.

Fresh silicate grout possesses very low resistivity due to its high ionic strength and dissolved Na⁺–silicate species. As gelation progresses, resistivity gradually increases because polymerization reduces the number of mobile ions and immobilizes part of the pore solution within the gel matrix (Pham et al. 2011). In laboratory monitoring, grout resistivity increased from 0.3 to 0.55 Ω m within 15 h, correlating directly with gelation and early-stage curing. These resistivity changes provide a quantitative proxy for gel formation and can be used to infer gelation kinetics and reaction progression in situ.

The early work of Komine (2000, 1997) demonstrated that ERT can track the spatial distribution of silicate grout in medium and fine sands with high accuracy. Grout continuity was strongly correlated with resistivity decreases. Komine’s laboratory tests showed that effective solidification requires a high proportion of pore space to be filled with grout: typically more than 85% in coarse sands, more than 60% in medium sands, and more than 40% in fine sands. These studies also showed that ERT could detect preferential flow paths, gravity-driven downward migration, and the onset of grout discontinuities long before mechanical testing or coring could confirm them.

Laboratory tank experiments by Pham et al. (2011) further validated the method for real-time monitoring. Cross-borehole bipole–bipole arrays produced the highest resolution, capturing both the advancing grout front and the evolution of resistivity during gelation. Synthetic modeling and inversion confirmed that resistivity tomograms closely matched the actual injected grout volume and geometry, even in relatively heterogeneous sands.

Despite its advantages, interpretation of ERT data requires careful consideration of electrode configuration, inversion artifacts, and the effects of pore-water conductivity. Elevated groundwater salinity can reduce resistivity contrasts and complicate inversion, while features smaller than the electrode spacing may remain unresolved. Even with these limitations, ERT remains a robust, field-ready method for characterizing and monitoring silicate grouting. Its sensitivity to grout continuity, spatial variability, and gelation kinetics makes it particularly valuable for quality control in permeation grouting and for diagnosing long-term changes resulting from environmental conditions or degradation. Future research should integrate ERT with hydrogeophysical modeling and reactive transport simulations to improve the prediction of grout evolution, especially under dynamic groundwater flow conditions.

## Microstructure Evolution

The microstructure of sodium silicate gels evolves dynamically during gelation and aging, and these transformations critically govern the hydraulic, mechanical, and durability characteristics of grouted soils. Evidence from SEM, cryo-SEM, NMR, and MIP consistently shows that gels transition from loosely aggregated colloidal networks to progressively densified, interconnected Si–O–Si frameworks. The pore architecture, its size, connectivity, and evolution is strongly governed by pH, relative density, salinity, temperature, and confinement by sand grains.

**Fig. 6:**
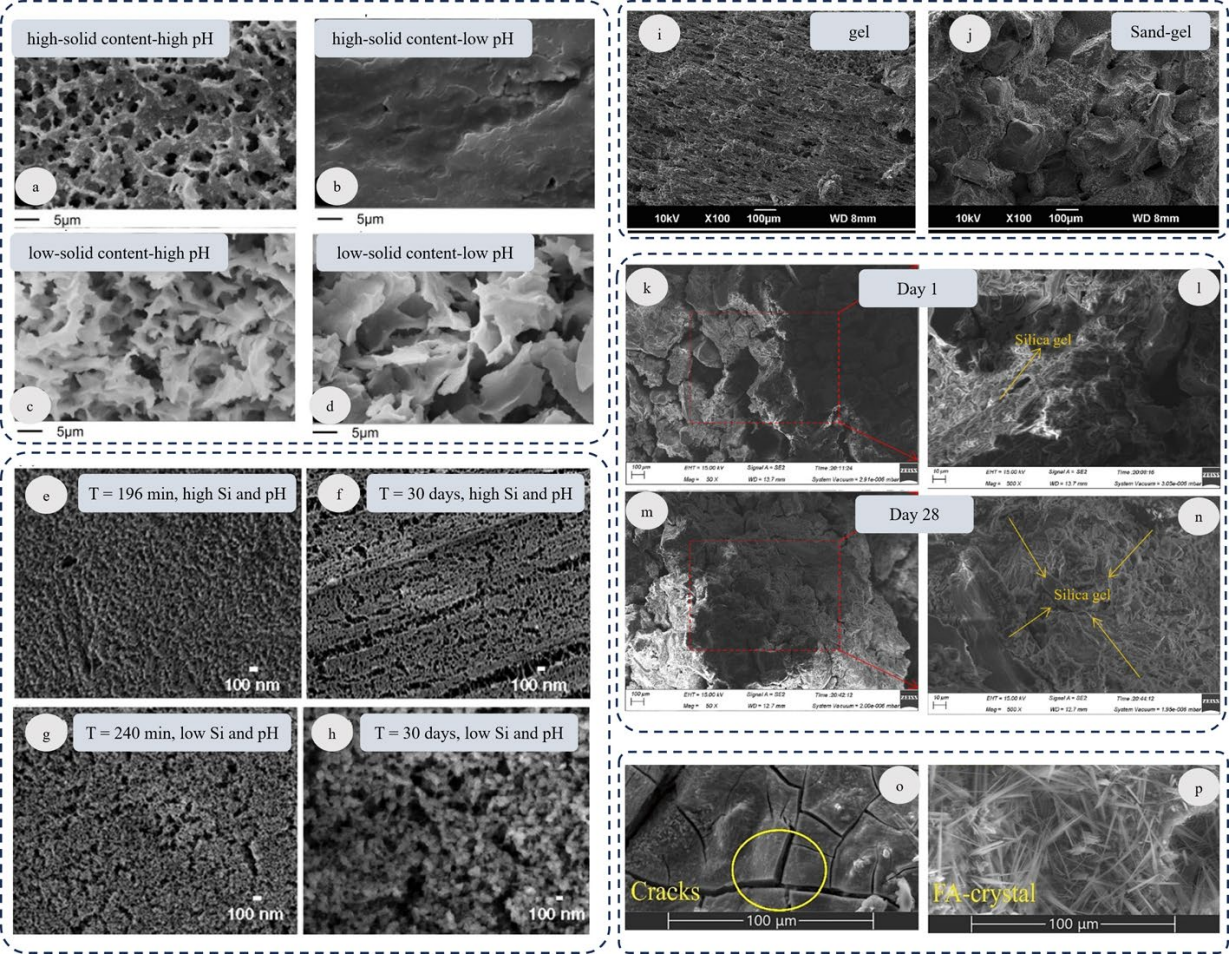
Representative SEM and cryo-SEM images illustrating the microstructure of silica gels and sand–gel composites: **a**–**d** SEM image of freeze-dried silica gels 1 h after gelation using phosphoric acid as the hardener (reproduced with permission from (Matinfar et al. 2025). © Elsevier, 2025). Panels **a** and **b** show high-solids gels (hard-gel morphology), whereas c and d show diluted formulations (soft-gel morphology). **a** and **c** at high pH; **b** and **d** at low pH. **e**–**h** Cryo-SEM images of HCl-hardened silica gels immediately after gelation **e**, **g** and after 30 days of aging **f**, **h**. Panels e and f correspond to gels with 3 mol/L Si at pH 10.67; **g** and **h** correspond to gels with 2.26 mol/L Si at pH 10.50 (reproduced with permission from (Tognonvi et al. 2011). © Springer Nature, 2011). **i**–**j** SEM images of silica gel and silica–sand mixtures cured with diacid ester hardeners (reproduced with permission from (Cui et al. 2022). © Elsevier, 2022). **k**–**n** SEM images of sand cured using sodium silicate and phosphoric acid. Panels **k** and **m** show microstructures after 1 day and 28 days of curing, respectively, while l and n present the corresponding magnified regions highlighting silica-gel (reproduced with permission from (Wang et al. 2023c). © Elsevier, 2023). **o**–**p** SEM images of silica gels cured by evaporation at 100 °C, showing formation of acicular and columnar SiO_2_ crystals during high-temperature curing (reproduced with permission from (Xu et al. 2023). © Elsevier, 2023)

Immediately after gelation, pure sodium silicate gels consist of flocculated aggregates of nanometric primary particles (5–20 nm) and loosely connected colloidal clusters (100–500 nm). Their morphology differs strongly with pH. Acidic gels (pH 2–5) exhibit compact fused-sphere structures with pores < 0.5 μm, while basic gels (pH 9–10.5) form open, mesh-like networks with large pores (1–20 μm), as shown in panels a and b in Fig. 6 (Matinfar et al. 2025; Matinfar and Nychka 2024). Dilution shifts speciation toward Q^1^–Q^2^ units, producing more flexible networks with larger and irregular pore geometries (panel c and d) (Matinfar et al. 2025). Cryo-SEM observations from Tognonvi et al. (2011) reveal that sodium-silicate gels undergo profound microstructural evolution during ripening, controlled by dissolution–reprecipitation processes. Immediately after gelation, both soluble (High pH and Si) and irreversible (low pH and Si) gels consist of homogeneous networks of nanometric silicate aggregates distributed in a fine porous matrix shown in panel e and g, respectively. During aging, soluble gels exhibit extensive pore coalescence and structural collapse, producing anisotropic macropore bands and weakly consolidated, water-soluble residues (Fig. 6f). In contrast, irreversible gels show more limited pore enlargement and retain a continuous, monolithic skeleton (Fig. 6h), consistent with the formation of an insoluble silica-rich phase (NaSi_12.66_O_25.82_) with high surface area (~ 280–290 m^2^/g) (Tognonvi et al. 2011).

Silicate gels formed within sand display fundamentally different microstructures. Mineral surfaces catalyze the condensation of the gel, producing much denser gel films than free gels. Instead of forming self-supporting networks, gels adhere to sand grains, forming thin interfacial coatings and intergranular bridges. SEM images show that these coatings fill micro voids, and create continuous cementing contacts (Fig. 6j) (Chen et al. 2023; Cui et al. 2022). These interfacial films are typically more condensed and less porous due to surface-catalyzed polymerization on quartz and aluminosilicates (panel i vs. j). The presence of reactive minerals introduces additional microstructural pathways. Carbonates react with silicate solutions to form Ca–silicate hydrates, producing compact crystalline layers that further reduce porosity (Guo et al. 2024).

Pore-size distributions in grouted sands transition from wide, bimodal distributions typical of sandy soils to narrower, refined systems dominated by silica-filled micropores. MIP analyses show shifts from 10 to 100 μm pores to 1–10 μm or finer depending on relative density and gel content (Chen et al. 2023). SEM analysis of the silicate grouted sand shows the progressive pore filling by aging of the silica gel which results in the decrease in porosity and increase of the cementation (panels k–n), which is mostly due to the syneresis of the silica and dissolution of the Microcline to form silica gel as proved by XRD (Wang et al. 2023c). During curing and aging, porosity continues to evolve through dehydration, condensation, microcracking, and mineral precipitation. In thick gel films, over-dehydration may induce microcracks (panel o), reducing bonding and increasing long-term permeability. Elevated temperatures accelerate polymerization and can lead to formation of acicular or columnar SiO_2_ crystals (panel p), strengthening the matrix initially but potentially reducing ductility at later stages (Xu et al. 2023).

There is no clear consensus in the literature regarding the impact of aging on the microstructural evolution of silica gel, as it is influenced by multiple interconnected processes including syneresis, dissolution–precipitation, and curing conditions. Moreover, most microstructural observations of grouted sand have been obtained from silicate systems cured by evaporation, which does not fully represent conditions in silicate grouting. Future research should therefore investigate the microstructural development of silica gel confined within sand, ideally using Cryo-SEM to prevent pore collapse during imaging. Understanding pore-scale evolution under realistic conditions is essential for predicting long-term durability in silicate-grouted soils.

## Permeability Reduction and Strength Enhancement

**Fig. 7:**
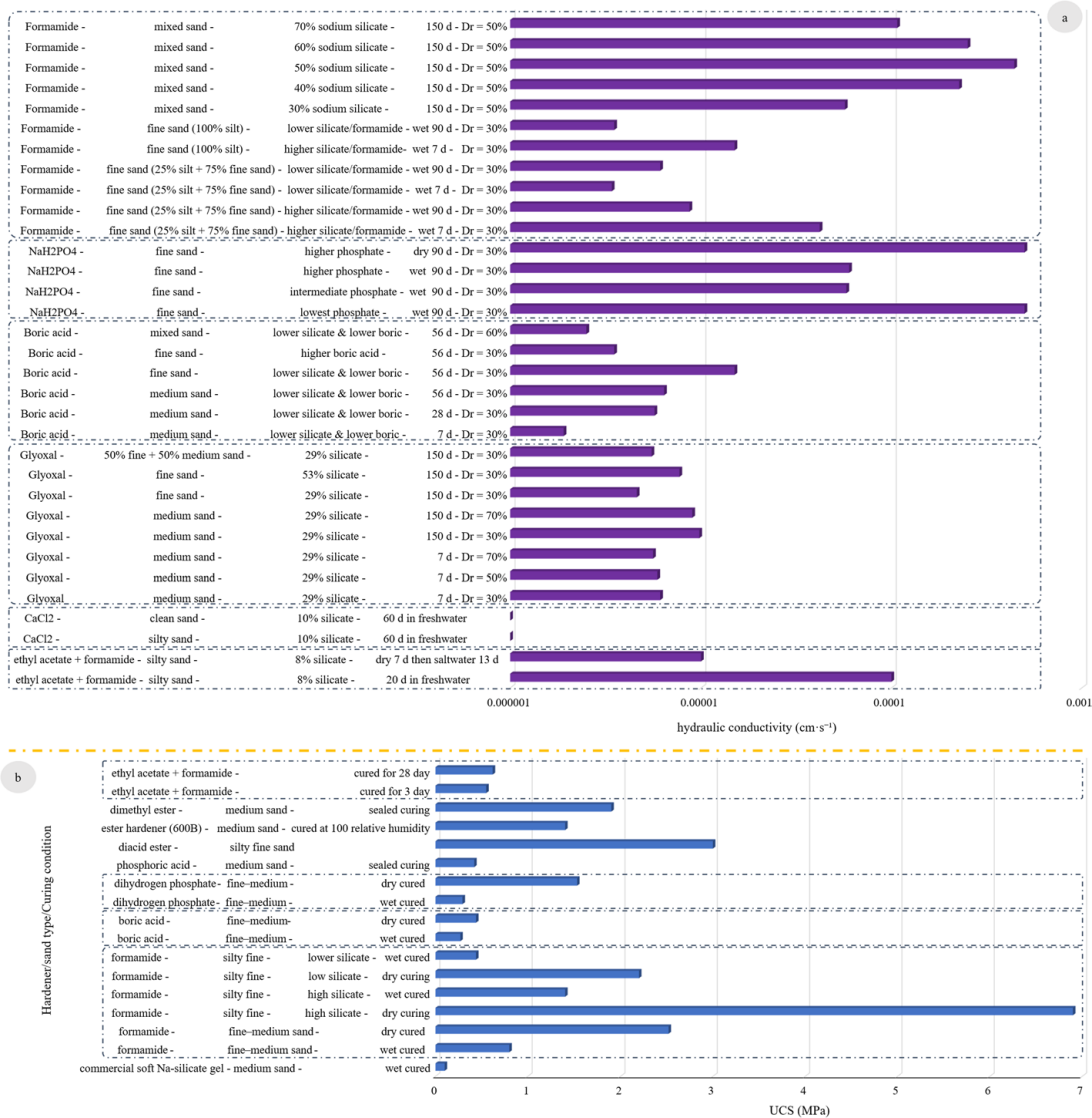
**a** Summary of post-grouting hydraulic conductivity values reported for silicate–treated sands, silty sands, and silt–sand mixtures across the literature. Each horizontal bar represents the range of measured hydraulic conductivity (k, in cm·s⁻^1^, log scale) for a unique combination of soil gradation, relative density (Dr), silicate concentration, and hardener type (organic or inorganic) (Avci et al. 2022, 2021; Avci 2017; Elektorowicz et al. 2008; Mollamahmutoğlu et al. 2017; Mollamahmutoğlu and Avci 2016). **b** Summary of unconfined compressive strength (UCS) values reported for silicate grouts across different soil types, hardener chemistries, silicate contents, and curing regimes (wet vs. dry) (Avci et al. 2021; Chen et al. 2023; Gonzalez and Vipulanandan 2007; Littlejohn and Mollamahmutoglu 1994; Mollamahmutoğlu et al. 2021; Mollamahmutoğlu and Avci 2020, 2016; Porcino et al. 2012; Wang et al. 2023c)

Permeation grouting with sodium silicate systematically reduces the permeability of sandy or gravely soils by several orders of magnitude whilst enhancing cohesion within the soils, through silica-gel bridges and reaction products at grain contacts. The magnitude and persistence of these improvements depend on grout chemistry (silicate content, relative density, hardener type, modulus), soil grading and density, curing conditions (temperature, wet vs. dry), and the long-term balance between gel dehydration, syneresis, and dissolution as listed in Fig. 7.

From a hydraulic perspective, most laboratory and field studies report permeability (Permeability values refer to hydraulic conductivity k (cm s^−1^)) reductions of 2–5 orders of magnitude in sands and silty sands. Classic permeation tests with organic-hardened silicate systems (formamide, glyoxal, esters) showed hydraulic conductivity reductions of fine to medium sand, having an initial hydraulic conductivity value of 10^−3^ –10^−1^ c s^−1^, to values in the range of 10^−6^ – 10^−5^ cm s^−1^, even at relatively low grout content (Avci 2017; Littlejohn et al. 1997; Mollamahmutoğlu and Avci 2016). Comparable reductions were obtained with inorganic hardeners such as sodium dihydrogen phosphate and boric acid, where permeability typically decreased to 10^−5^ – 10^−6^ cm s^−1^ and remained in that range during the first months of curing (Mollamahmutoğlu et al. 2021, 2017). In more refined systems developed for tunnel and metro applications, silicate–phosphate and diacid-ester grouts achieved permeabilities on the order of 10^−7^ – 10^−8^ cm s^−1^ in treated sand columns and model tests, while maintaining sufficient injectability for poorly graded silty sands (Cui et al. 2022; Wang et al. 2023c). The compiled data in panel a of Fig. 7 confirm that the lowest permeabilities are consistently obtained in fine or silty sands, where small pore throats and high specific surface favor uniform gel deposition and confinement of the gel network.

The mechanisms of permeability reduction are broadly consistent across studies. In quartz sands and silty sands, sodium silicate gels precipitate within pore spaces and as coatings on grain surfaces, forming a continuous network that partially or fully fills the pore skeleton (Chen et al. 2023; Porcino et al. 2012). SEM and MIP analyses show progressive infilling of macropores, coarsening of the effective particle size, and a shift in the pore-size distribution toward mesopores and micropores as curing proceeds (Chen et al. 2023; Porcino et al. 2012). Because pore throats have the highest ratio of solid surface area to pore volume, they act as preferential sites for silicate deposition and gel growth. As a result, even limited precipitation at these constrictions produces significant reductions in hydraulic conductivity, with permeability loss governed primarily by pore-throat narrowing and partial loss of pore connectivity.

Long-term permeability trends are strongly influenced by syneresis and gel shrinkage. Column and tank tests consistently show that permeability decreases sharply immediately after grouting, then increases slightly (typically by ~ 5–20%) over months to years as shrinkage, microcracking and minor dissolution occur, before stabilizing at a quasi-steady value that is still several orders of magnitude lower than the untreated soil (Avci 2017; Bodocsi and Bowers 1991; Elektorowicz et al. 2008; Krizek and Spino 2000; Mollamahmutoğlu et al. 2017; Mollamahmutoğlu and Avci 2016). For example, phosphate-silicate grouts in sand showed permeability reductions of 1–5 orders of magnitude, with the lowest values in fine sands and high-phosphate mixes; wet-cured specimens retained permeability (k) in the 10^−6^–10^−5^ cm s^−1^ range despite some strength loss due to syneresis (Mollamahmutoğlu et al. 2021). Boric-acid silicate grouts in river sand exhibited similar 2–3 order-of-magnitude reductions, with a modest increase in k over 150 days linked to gel shrinkage (Mollamahmutoğlu et al. 2017). Glyoxal-modified silicate grouts achieved k ≈ 10^−6^ cm s^−1^ but showed a gradual permeability increase over months as matrix shrinkage progressed (Avci 2017).

Groundwater chemistry strongly controls hydraulic performance. Elektorowicz et al. 2008 (Elektorowicz et al. 2008)showed that CaCl₂-modified grouts maintained permeabilities of ~ 10^−6^ cm s^−1^ for at least 60 days in both fresh and saltwater, whereas organic-hardened grouts degraded significantly, with k increasing from 10^−6^ to 10^−4^ cm s^−1^ in freshwater. In the same study, carbonate film formation in mineralized water further reduced leaching and protected the gel. In contrast, experiments on sand columns grouted with hard gels revealed that immersion in saline water can increase permeability, which was attributed to gel shrinkage caused by dehydration or ion exchange at high salinity (Elektorowicz et al. 2008).

In parallel, silicate grouting provides substantial strength enhancement, primarily by introducing apparent cohesion while largely preserving the friction angle. Lightly cemented sands treated with low-solids or “soft” silicate gels typically reach unconfined compressive strengths (UCS) below 100–200 kPa, with friction angles close to those of the untreated soil (Hatzignatiou et al. 2014; Porcino et al. 2012). Conventional ester- and formamide-based systems in medium sands commonly achieve UCS values of 0.3–1.5 MPa after 7–28 days, depending on silicate content, relative density and curing conditions; air-dried specimens are generally 2–4 times stronger than wet-cured ones due to dehydration-induced matrix tightening(Gonzalez and Vipulanandan 2007; Littlejohn et al. 1997; Mollamahmutoğlu and Avci 2016; Mollamahmutoglu and Littlejohn 1997). In formamide-silicate grouted silty sands, air-dried UCS values up to 6.9 MPa have been reported, while corresponding wet-cured strengths decreased by up to 56% over 90 days (Avci et al. 2022). Phosphate-silicate grouts in sand showed air-dried UCS between 740 and 1529 kPa versus 49–293 kPa under wet curing, again highlighting the strong influence of moisture content (Mollamahmutoğlu et al. 2021).

More heavily cemented systems can reach much higher strengths. NaOH-modified silicate grouts and high-relative density formulations in sand and loess have produced UCS values of 3–9 MPa, particularly under combined room-temperature and elevated-temperature curing (Chen et al. 2023; Guo et al. 2024; Xu et al. 2023). Diacid-ester-based grouts in silty fine sands achieved compressive strengths of 1.6–3.0 MPa and retained more than 60% of their strength after 28 days in both air and water, outperforming phosphate-modified silicate systems in terms of durability (Cui et al. 2022).

The micromechanical origin of strength enhancement has been clarified by SEM and NMR studies. At the grain scale, silica gels precipitate as thin films and menisci around sand particles, forming continuous bridges across contacts and partially filling small pores (Chen et al. 2023; Porcino et al. 2012; Xu et al. 2023). In carbonate sands and loess, Ca-silicate hydrates and mixed Ca–Na–silicate products further stiffen and roughen contacts, reducing slip and grain crushing (Guo et al. 2024; Salehzadeh et al. 2012). Interface tests and composite models show that three coupled components control the macroscopic strength of grouted sand: (i) cohesive strength of the gel matrix, (ii) adhesive bond strength at the sand–gel interface, and (iii) the fabric and porosity of the sand skeleton (Ata and Vipulanandan 1998; Kaga and Yonekura 1991; Vipulanandan and Krizek 1986). Strength scales positively with grout tensile strength, grout/void ratio and specific surface area, and can be predicted reasonably well using Eq. (4). Where *q*_u_ is the UCS of the grouted sand,* q*_u*,*gel_ is the UCS of the pure gel, and *A*, *B*, and *n* are the parameters depending on the porosity, surface area, and density (Kaga and Yonekura 1991). linking grouted-sand UCS to pure grout strength and sand porosity (Ata and Vipulanandan 1998; Kaga and Yonekura 1991). Most studies report that the friction angle φ′ remains close to that of the untreated sand, whereas the apparent cohesion c′ increases markedly and governs the enhanced dilatancy, liquefaction resistance and cyclic stability of treated soils (Guo et al. 2024; Hatzignatiou et al. 2014; Porcino et al. 2012; Vipulanandan and Ata 2000).

4 $$q_{{\mathrm{u}}} = A\left( {q_{{{\mathrm{u}},{\mathrm{gel}}}} + B} \right)^{{\mathrm{n}}}$$

Taken together, the literature indicates that sodium-silicate grouting can reliably deliver large permeability reductions and significant strength gains in sandy and silty soils, provided that gel composition, relative density and hardener type are matched to the grain-size distribution, groundwater chemistry and service conditions.

## Summary and Conclusion

Silicate grouting is a multi-scale, multi-phase process involving three temporal stages (injection, gel formation, and aging) and four interacting components: gel, water, air, and soil. It enables construction in complex hydrogeological settings by forming low-permeability and strength-enhancing barriers within unconsolidated soils. However, its efficiency and long-term performance depend on a combination of engineered parameters (e.g., viscosity, gelation time, composition) and environmental conditions (e.g., soil permeability, groundwater chemistry, salinity, and organic content).

During the injection stage, hydrodynamic dispersion dilutes the grout and reduces gel saturation. This effect is more pronounced for soft gels with lower silica content. Injection efficiency can be improved by minimizing dilution through optimized injection spacing, volume, and timing, or by engineering formulations that gel at lower dilution ratios. Improving injection efficiency offers several benefits, including reducing environmental impact by minimizing leaching into groundwater, lowering the risk of well clogging at construction sites and nearby subsurface energy systems like ATES, and reducing material costs per injection.

The aging stage remains the least understood yet most critical phase. Over time, silicate gels undergo syneresis, swelling, and chemical degradation that alter permeability and mechanical strength. These changes depend on environmental factors such as groundwater composition, salinity, and contaminant levels, as well as interactions with soil minerals and organic matter. Short-term aging may cause well clogging, intermediate stages can induce leakage through the grout layer, and long-term degradation threatens subsurface structures and energy systems. A quantitative understanding of these time-dependent processes is essential for predicting performance and ensuring durability.

Future research should focus on the following areas to achieve durable and sustainable application of silicate grouting:

- Quantifying the impact of environmental parameters (permeability, dispersivity, groundwater composition, and temperature) on grout behavior to guide the design of site-specific formulations.
- Developing constitutive relationships for key physical and transport properties of silicate grout, including density, viscosity, and thermal conductivity, as functions of temperature, time, and component concentrations, to support accurate predictive modelling of the injection stage.
- Establishing and validating numerical approaches capable of predicting temperature evolution during injection, gelation, and aging, since temperature is one of the dominant factors governing reaction kinetics, syneresis, strength gain, and long-term stability.
- Improving injection efficiency through refined flow modeling, optimized injection spacing and timing, and adaptive viscosity control to increase gel saturation while minimizing environmental footprint and cost.
- Silicate grouts interact dynamically with contaminants, organic matter, and minerals. Understanding these interactions under realistic field conditions is crucial for maintaining grout stability and mitigating pollutant migration.
- Understanding the physical and chemical mechanisms driving gel degradation under flowing groundwater and developing predictive, time-dependent models.
- Quantifying leaching rates of dissolved silica and associated ions, and assessing their influence on groundwater quality and adjacent systems, is vital for sustainable design and regulatory compliance.
- Integrating geophysical monitoring, particularly ERT, with grouting operations to track the formation, continuity, and heterogeneity of the grouted layer, enabling targeted reinjection in insufficiently treated zones and increasing the overall success rate of field applications.
- Developing silicate grout formulations with near-neutral pH to minimize chemical disturbances to surrounding wells, aquifers, and geothermal systems, reducing clogging risks and improving compatibility with sensitive subsurface environments.

Addressing these challenges demands interdisciplinary research combining geotechnical engineering, hydrogeology, and materials science. Collaborative efforts between researchers and industry practitioners are needed to develop predictive models that link chemical reactions, flow behavior, and long-term performance. By advancing understanding of the coupled physical and chemical processes governing silicate grouting, future applications can achieve higher construction efficiency, environmental safety, and long-term sustainability in subsurface engineering.

## Supplementary Information

Below is the link to the electronic supplementary material.

Supplementary file1 (PDF 459 kb)

## Data Availability

No datasets were generated or analysed during the current study.

## Declarations

### Generative AI and AI‑Assisted Technologies

Generative AI and AI-assisted technologies have been used to refine language, and ensure readability of the paper.
