# Supplementary material for: Sodium Silicate Grouting: Mechanisms, Environmental Impacts, and Research Directions
Source: Transp Porous Media. 2026 Jun 19;153(6):78. doi: 10.1007/s11242-026-02323-3 (PMC13282353; doi:10.1007/s11242-026-02323-3)
Supplement: Supplementary file 1 — Supplementary file1 (PDF 459 kb) [file 11242_2026_2323_MOESM1_ESM.pdf]

Supplementary material for:

Sodium silicate grouting: mechanisms, environmental impacts, and research directions

Mohammad Valibeknejad [a*], Thomas Sweijen [a], Alraune Zech [a], Julian Quodbach [b], Noushine Shahidzadeh [c], Mariette Wolthers [d], Amir Raoof [a]

[a] Environmental Hydrogeology Group, Department of Earth Sciences, Utrecht University, Utrecht, the Netherlands

[b] Division of Pharmaceutics, Utrecht Institute for Pharmaceutical Sciences, Utrecht University, Utrecht, the Netherlands

[c] Institute of Physics, University of Amsterdam, Amsterdam, the Netherlands

[d] Geochemistry Group, Department of Earth Sciences, Utrecht University, Utrecht, the Netherlands

[*] Corresponding author at: m.valibeknejad@uu.nl

Table. S1 key studies that underpin current understanding of sodium–silicate grouting. The table documents the diversity of grout formulations (inorganic and organic hardeners), test methodologies, soil types, and environmental conditions examined in the literature. For each study, the principal mechanistic, chemical, hydraulic, or mechanical insights are summarized. This consolidated dataset provides the foundation for the comparative analysis presented in the main manuscript and highlights the evolution of silicate grouting research over the past decades.

Author(s), year	Grout type / hardener	Test condition	Key findings
Hurley, C.H., & Thornburn, T.H. (1966) (Hurley and Thornburn 1971)	Sodium silicate with hardeners such as $CaCl_2$, $MgCO_3$, $NaHCO_3$, $NaAlO_2$, or Na_2SiO_3–$MgCO_3$ systems.	Review on silicate soil stabilization, grouting, and additive use with cement, lime, and fly ash.	• Mechanisms: Na_2SiO_3 reacts with acidic or multivalent cations (Ca^{2+}, Mg^{2+}, Al^{3+}) to form amorphous silica gels or metal silicate networks; these provide cohesion and permeability reduction.<br>• Gel behavior: Gels undergo syneresis, desiccation, and dissolution, losing 20–60% water in 2 months and up to 25% of volume.<br>• Soil–gel interaction: Favorable with sandy and low-plasticity soils; poor with montmorillonite-rich clays or soils containing organics.<br>• Durability: Alkaline groundwater and freeze–thaw cycles degrade gels.<br>• Salinity/organic effects: Salts shorten gel time; organics can prevent gelation entirely.
Visser, J.H.M. (2018) (Visser 2018)	Synthetic alkali-silica gels formed via condensation of silicic acid anions under different cation environments (Na^+, K^+, Ca^{2+}, Mg^{2+}, etc.).	Review of the gel formation and aging under varying cation environments.	• Gel formation is a condensation reaction between silicic acid anions, not ionic binding. Cations act as charge-screening agents facilitating condensation.<br>• Cation Gelling Strength (CGS) introduced, gelation rate inversely proportional to CGS; divalent cations (Ca^{2+}, Mg^{2+}) cause faster gelation at lower concentrations than monovalent ions (Na^+, K^+).<br>• Hydration effects: small, strongly hydrated ions (Na^+, Li^+) reduce charge neutralization → weaker gels.<br>• Swelling mechanism: Osmotic water uptake occurs due to concentration differences between gel interior and exterior; swelling potential decreases with higher Ca/Si ratio and increased crosslinking.<br>• Aging: Continued condensation converts $Q_2 \rightarrow Q_3 \rightarrow Q_4$ bonds, causing shrinkage and syneresis (expulsion of water).<br>• Cation exchange ($Na^+ \rightarrow Ca^{2+}$, Mg^{2+}) during aging reduces swelling potential and increases stiffness.<br>• Precipitation vs. gelation: High Ca^{2+} or Mg^{2+} concentrations promote precipitation of dense calcium silicate phases rather than gelation.<br>• Defines equilibrium behavior and transitions between swelling, syneresis, and dissolution.

Matinfar, M. & Nychka, J.A. (2023) (Matinfar and Nychka 2023)	Sodium silicate solutions in various compositions (SiO_2/Na_2O ratio = 1.6–3.75), with gelation induced by acidification, temperature, or salt addition (Na^+, K^+, Ca^{2+}, Mg^{2+}).	Comprehensive review combining 70 years of experimental and modeling studies (NMR, Raman, SAXS, IR, DLS, MD, QM, Monte Carlo).	• Structure: Sodium silicate is a dynamic mixture of monomeric, oligomeric, and colloidal silicate species (Q^0–Q^4). Polymerization increases with silica content and decreases with temperature and pH.<br>• Gelation: Occurs via nucleation, aggregation, and network formation; initiated by protonation or cation exchange ($Na^+ \rightarrow H^+/Ca^{2+}/Mg^{2+}$). Acidic and neutral gels form dense silica networks; basic gels form more porous structures.<br>• Gelation kinetics: Gel time decreases with higher silica content, lower pH, and increased ionic strength; divalent cations (Ca^{2+}, Mg^{2+}) drastically shorten gel time.<br>• Syneresis: Driven by continued Si–O–Si condensation, leading to shrinkage and water expulsion. Rate increases with temperature, ionic strength, and divalent cation presence.<br>• Aging: Gels evolve through Ostwald ripening and condensation, forming stiffer, less permeable networks.<br>• Molecular modeling: Confirms multi-step condensation via penta-coordinated intermediates; shows cation effects on stability and polymerization; supports experimental findings.<br>• Unresolved issues: Effects of aging, long-term structure of acidic vs. basic gels, and the interplay of Na^+, OH^-, and water during syneresis.
Matinfar, M. & Nychka, J.A. (2025) (Matinfar and Nychka 2025)	Sodium silicate solution gelled using boric acid (BA) or phosphoric acid (PA) as acid initiators.	Raman spectroscopy (real-time, 2-min intervals) and SEM characterization; comparison of acidic (pH 3–5) vs. basic (pH 9–10.6) gels.	• Gelation mechanism: Acid-initiated sol–gel transition occurs mainly via particle agglomeration (secondary bonding), hydrogen and electrostatic interactions, not new Si–O–Si bond formation, indicating physical rather than chemical gels.<br>• Structure: Basic gels dominated by Q^3 (53–80%), Q^2 (10–17%), Q^0 (5–34%); Acidic gels dominated by Q^2 (62–80%) and Q^0 (20–38%).<br>• pH effect: Acidic gels → smaller, denser pores (compact network); Basic gels → larger, open pores (loose network).<br>• Polymerization trends: Acidic environment → hydrolysis and depolymerization ($Q^3 \rightarrow Q^2/Q^0$); Basic environment → condensation and polymerization ($Q^1 \rightarrow Q^3$).<br>• Agglomeration: Basic gels form large, loose secondary particles (pores ~5 μm); Acidic gels form small, compact ones (<550 nm).<br>• Dilution effect: Higher water ratios lead to depolymerization ($Q_3/Q_2 \rightarrow Q_1/Q_0$) due to Na^+ dissociation and Si–O–Si bond hydrolysis.<br>• Mechanistic insight: Confirms that silica gelation from sodium silicate can occur without new covalent bonding, controlled primarily by electrostatic and hydrogen-bond interactions.
Matinfar, M., Elias, A., & Nychka, J.A. (2025) (Matinfar et al. 2025)	Sodium silicate (SiO_2/Na_2O = 2.57); acid-initiated using boric acid (BA) and phosphoric acid (PA).	Compression tests with real-time area monitoring to obtain true stress–strain curves; supplemented by SEM imaging after freeze-drying at 5 min and 60 min post-gelation.	• Aging & Syneresis: Both acidic and basic gels strengthen over time (5 → 60 min) due to continued condensation and water expulsion. Strength increases up to 7 times for concentrated acidic gels.<br>• Mechanical behavior: Identified three distinct behaviors: (i) brittle fracture (acidic 1:1), (ii) plastic plateau (acidic 1:3, 1:5; basic 1:5), and (iii) continuous strain hardening (basic 1:1, 1:3).<br>• Strength evolution: Acidic 1:1 gels = 53.6 MPa (brittle, small pores); Basic 1:3 gels = 38 MPa (ductile, large pores). Dilution reduces strength but increases ductility and pore size (up to 20 μm).<br>• Aging mechanism: Strength increase governed by ongoing Si–O–Si condensation and network densification. Acidic gels gain strength mainly from bond formation, basic gels from syneresis and shrinkage.<br>• Syneresis dependence: More pronounced in basic gels due to higher pore water mobility; reduced at lower silica concentrations.<br>• Fractography: Revealed radial, circumferential, and splitting cracks; brittle gels fragment under stress, ductile gels exhibit crack blunting.<br>• Microstructure: Basic gels → open mesh networks (3–20 μm pores, ductile); Acidic gels → fused-sphere morphology (1–3 μm, brittle). Over time, both densify; basic gels show wall thickening, acidic gels glass-like compaction.<br>• Stress–strain accuracy: Engineering stress overestimated true strength by 25–60%, underscoring the need for true stress–strain analysis.

Matinfar, M., & Nychka, J.A. (2024) (Matinfar and Nychka 2024)	Sodium silicate (SiO_2/Na_2O = 2.57); acid-initiated with boric acid (BA) and phosphoric acid (PA).	Tube inversion test for gel time; UV–Vis spectroscopy and Tyndall effect for optical monitoring; SEM for microstructure.	• Gelation kinetics: In the basic region, gelation time follows an exponential relation with pH; in the acidic region, a third-order polynomial. Peak gelation delay at pH ≈ 2.<br>• Controlling gelation: Gelation kinetics mainly depend on pH and waterglass concentration, not acid type.<br>• Microstructure: Acidic gels, smaller SiO_2 particles and aggregates (dense, glass-like, pores <550 nm); Basic gels, larger aggregates and pores (~5 µm), opaque.<br>• Optical properties: Acidic gels → high transmittance (>90%); Basic gels → opaque (<10%). Transparency differences directly reflect cluster and pore size.<br>• Concentration effect: Dilution increases gel time and pore size; in acidic gels, transparency decreases with dilution; in basic gels, transparency increases.<br>• Kinetics via UV–Vis: Derivative of transmittance curve detects onset of gelation ≈ 8 min, consistent with 10 min full gelation.<br>• Syneresis: Light transmittance continues changing post-gelation → ongoing densification and water expulsion.<br>• Modeling: Empirical relations enable predictive control of gel time and microstructure.
Hatzignatiou, D.G., & Giske, N.H. (2018) (Hatzignatiou and Giske 2018)	sodium silicate / NaCl, HCl, HNO_3, HCOOH, and urea.	Tests in chalk and sandstone cores (20–90 °C).	• Gelation time decreases with increasing Na^+, Ca^{2+}, temperature, and silicate concentration. Dissolved Ca^{2+} from chalk strongly accelerates gelation (~5 times).<br>• Adding sand as filler increased gel strength (>180 bar); polymers (HPAM, xanthan, diutan) often weakened gels or caused syneresis.<br>• Alkaline solutions (NaOH, KOH, ≥2 M) fully dissolved gels within hours; dissolution rate increased with alkalinity and temperature.<br>• Injected silicate partially reacted with chalk, producing localized Ca-silicate gel layers at the fracture interface.
Nasr-El-Din, H.A., & Taylor, K.C. (2005) (Nasr-El-Din and Taylor 2005)	Sodium silicate (SiO_2/Na_2O = 3.29) with urea as an activator.	Bulk gelation and coreflood experiments using limestone (calcite) cores.	• Urea hydrolysis generates $NH_4^+ + OCN^-$, which reduces the dielectric constant of solution → induces gelation without significant pH drop.<br>• Gelation occurs only at T > 70 °C; rate increases with urea concentration but excessive urea causes syneresis and shrinkage.<br>• NaCl (≤ 3 wt%) and $CaCl_2$ (≤ 0.08 wt%) tolerated, both accelerate gelation but higher concentrations cause precipitation (Ca–silicate formation).<br>• Coreflood tests showed > 1000 times permeability reduction and gel stability up to 56.6 MPa/m pressure gradient.<br>• Minimal dissolution or leaching of silica (effluent Si ≈ 25–30 mg/L).<br>• Preflush with low-salinity brine (0.5 wt% NaCl) required to avoid Ca–Mg precipitation and ensure injectivity.
Wijnen, P.W.J.G., Beelen, T.P.M., de Haan, J.W., Rummens, C.P.J., & van Santen, R.A. (1989) (Wijnen et al. 1989)	Amorphous silica gel dissolved in LiOH, NaOH, KOH, RbOH, CsOH aqueous solutions.	Dissolution kinetics monitored via ^{29}Si-NMR and β-silicomolybdate analysis; ambient conditions (25 °C, 1 atm).	• Dissolution rate increases in the order (LiOH ≈ CsOH) < (RbOH ≈ NaOH) < KOH, showing maximum reactivity with KOH.<br>• Dissolution involves formation of monomeric $Si(OH)_4$, which oligomerizes into dimers, trimers (linear/cyclic), and multicyclic Q^3 species.<br>• Alkali cations influence silica polymerization and species distribution (Q^2/Q^3 ratio), K^+ promotes the most polymerized silicate structures.<br>• Hydroxyl anions catalyze dissolution by increasing Si coordination (>4), weakening Si–O–Si bonds.<br>• Cation hydration structure explains reactivity: Li^+, Na^+ (strongly hydrated) → slower dissolution; K^+, Rb^+, Cs^+ (water structure breakers) → faster dissolution.<br>• The composition of dissolved silicate species (degree of polymerization and structural symmetry) depends on the specific alkali cation present.<br>• Overall, the study clarifies mechanisms of gel depolymerization and reprecipitation, linking them to pH, cation type, and hydroxide concentration.

Hashemi, S.J., Hormozi, F., & Mokhtari, R. (2023) (Hashemi et al. 2023)	Sodium silicate with citric acid (as gelling and ion-masking agent). In some cases, SiO_2 nanoparticles (15–20 nm) used as stabilizers and nucleation promoters.	Experiments in bulk solutions under controlled temperature (25–100 °C); evaluated gelation time, and nanoparticle stability.	• Developed a novel gelation control method based on the H^+ protection mechanism, citric acid pre-flush creates an H^+ layer around nanoparticles, stabilizing them and delaying gelation.<br>• Citric acid (0.05–0.1 wt%) masks Ca^{2+} and Mg^{2+} in brines, preventing premature gelation and precipitation.<br>• H^+-protected nanoparticles delay gelation by up to 14 % but act as nucleation sites once the H^+ layer breaks down, enhancing gel strength.<br>• Gelation time decreases with higher Na_2SiO_3 or citric acid, but increases with optimal nanoparticle concentration (0.15–0.3 wt%).<br>• Nanoparticles remain stable (no precipitation) between 20–100 °C.<br>• Proposed citric acid pre-flush protocol as a practical solution to prevent premature gelation under saline conditions.
Lian, X., Peng, Z., Shen, L., Qi, T., Zhou, Q., Li, X., Liu, G. (2021) (LIAN et al. 2021)	Low-modulus sodium silicate ($m \leq 1$).	Pure silicate solution.	• Electrical conductivity increases with temperature and silica concentration, but decreases with modulus; confirms strong electrolyte behavior.<br>• At > 50 °C, pre-desilication solution forms sodium aluminosilicate hydrate precipitates, lowering conductivity.<br>• Viscosity rises with silica content and decreases linearly with temperature; surface tension decreases with silica concentration and temperature, confirming that sodium silicate behaves as an oligomeric surfactant.<br>• Identified a critical SiO_2 concentration (≈ 44.7 g/L), below it, monomeric silicate ions dominate; above it, polymerization into dimeric and polysilicate ions reduces ion mobility.<br>• Pre-desilication (Al-bearing) solutions show higher viscosity and lower surface tension than pure sodium silicate due to formation of complex aluminosilicate ions.
Dimas, D., Giannopoulou, I., Panias, D. (2009) (Dimas et al. 2009)	Sodium silicate and sodium aluminosilicate gels; gelation by accelerated polycondensation (evaporation at 60 °C).	Pure gels studied as model systems for geopolymer binders.	• Sodium silicate gels are amorphous, with structure strongly dependent on SiO_2/Na_2O ratio.<br>• Low ratios (< 3.5) → more non-bridging oxygen atoms (chain-like Q^2, Q^1 units), high solubility and low hardness.<br>• High ratios (> 4.4) → crosslinked frameworks (Q^3–Q^4 units), hydrolytically stable and hard gels.<br>• Hydrolytic stability and hardness both increase exponentially with SiO_2/Na_2O ratio, practically insoluble above 4.4.<br>• Al incorporation (as $NaAlO_2$ or $AlCl_3$) increases crosslinking, leading to higher Q_4 content, reduced solubility, and improved mechanical properties.<br>• Water in gels exists mostly as physically bound and hydrogen-bonded water.<br>• Establishes link between gel structure (Q^n units), strength, and water resistance.<br>• Suggests that additives (Al, B, Fe, P, etc.) can lower the minimum SiO_2/Na_2O ratio required for stable, durable gels, key for cost reduction and performance optimization.
Berrier, Courtheoux, Bouazaoui, Capoen, Turrell (2010) (Berrier et al. 2010)	Sol–gel derived tetramethyl orthosilicate system; doped with trace metal cations (500 ppm) such as Na^+, K^+, Ca^{2+}, Cu^{2+}, Pb^{2+}, Nd^{3+}, and Al^{3+}.	Pure silica gels; doped xerogels studied via Raman spectroscopy, N_2 adsorption–desorption, and pH monitoring.	• Even trace (ppm-level) cation doping strongly influences gelation kinetics, pH evolution, and final gel porosity.<br>• Al^{3+}, Cu^{2+}, Pb^{2+}, Nd^{3+} retard gelation, forming dense microporous gels with ~37 Å pores; Na^+, K^+, Ca^{2+} accelerate gelation, producing mesoporous gels (70–90 Å pores).<br>• pH evolution mirrored catalytic effects: fast gelation at pH ≈ 7–8; retarded at low pH (2–3).<br>• Raman spectra showed "acid-like" dopants stabilize small oligomers, while "base-like" dopants promote interparticle condensation.<br>• Structural differences: Na^+ and K^+ doping broaden Si–O–Si angles, reducing ring strain and stabilizing larger pore networks.<br>• Porosity–structure link: Accelerating ions → open mesoporous structures; retarding ions → denser, microporous networks.<br>• Conclusion: ppm-level metal cations can fine-tune silica gel texture (porosity, surface area, density) without affecting overall chemical composition, explaining ion-dependent gel behavior relevant to silicate grout modification in saline or contaminated environments.

Pham, L.T., Hatzignatiou, D.G. (2016) (Pham and Hatzignatiou 2016)	Sodium silicate with NaCl activator.	Gel tested in rheometer and capillary tubes under controlled temperature and pressure to simulate fractured reservoirs.	• Developed a unified sol–gel transition time correlation considering silicate concentration, activator concentration, temperature, divalent ions (Ca^{2+}, Mg^{2+}), and dilution.<br>• Activator concentration (NaCl) had the strongest effect, higher activator levels greatly accelerated gelation.<br>• Higher silicate content also shortened gelation, but less strongly.<br>• Temperature increase (40–60 °C) halved gelation time every 10 °C rise (Arrhenius relation).<br>• Ca^{2+} and Mg^{2+} ions shortened gel time; Mg^{2+} caused precipitation above ~200 ppm.<br>• Dilution with distilled water delayed gelation, while saline water accelerated it.<br>• Derived yield stresses up to 606 Pa and elastic moduli up to 8520 Pa, showing gels can withstand >20 bar differential pressure in microfractures.<br>• Pressure increase delayed gelation due to enhanced silicate solubility.
Hatzignatiou, D.G., Helleren, J., Stavland, A. (2014) (Hatzignatiou et al. 2014)	Sodium silicate ($SiO_2/Na_2O = 3.4$) activated with HCl (2 M); contained trace Al^{3+} (31 ppm) and Ca^{2+} (20 ppm).	Quartz sand columns, fully water-saturated.	• Combined bulk gelation and dynamic coreflood experiments to calibrate silicate gelation kinetics for flow-zone isolation and CO_2 leakage mitigation.<br>• Gelation time decreases with higher temperature, salinity, HCl concentration, and Ca^{2+} content.<br>• Derived an empirical relationship for gelation time.<br>• Calcium not directly involved in reaction but accelerates gelation via ion exchange.<br>• At 55–64 °C, gelation occurred after 3–6 days; higher salinity led to faster gelation and stronger gels.<br>• CO_2 applicability: Silicate gels remain stable in mildly acidic (carbonic acid) conditions, making them suitable for CO_2 storage sealing.
Hatzignatiou et al., (2016) (Hatzignatiou et al. 2016)	Sodium silicate activated by NaCl	Grouting of artificially fractured cores	• Gel strength increased with temperature (40–90°C) and aging time, following a logarithmic trend.<br>• Core tests: reduced fracture permeability by up to 10^4–10^5 times, though minor shrinkage was observed over weeks.<br>• Calcium ions accelerated gelation exponentially; Ca^{2+} at 100–1000 ppm reduced gelation time by >90%.
Quarch, K., Kind, M. (2010) (Quarch and Kind 2010)	Sodium silicate solution mixed with H_2SO_4 (50%); acidic and alkaline gelation regimes studied.	Pure gel; rheological tests with cone–plate rheometer to monitor in situ gelation.	• Identified two pH regions of rapid gelation: strongly acidic (< pH 2) and neutral–slightly alkaline (pH 7–8); gelation inhibited near the isoelectric point (pH ≈ 2).<br>• Acidic gels were stronger (higher elastic modulus, G′) than alkaline gels at equal solids content.<br>• Ionic strength accelerates gelation (via double-layer compression) in alkaline region but has negligible effect on acidic gels.<br>• Temperature (15–35 °C) accelerates gelation without reducing final gel strength.<br>• Developed a semi-empirical model for gelation time.
Katoueizadeh, E., Rasouli, M., Zebarjad, S.M. (2021) (Katoueizadeh et al. 2021)	Sodium silicate with H_2SO_4 as hardener.	Pure gel, tested using rheometer under shear rates 1–200 s^{-1} and frequencies 0.1–0.5 Hz.	• Gels exhibited non-Newtonian, shear-thinning (pseudoplastic) behavior; viscosity decreased with shear rate and time (thixotropic response).<br>• Mizrahi–Berk model best fitted data among six tested rheological models.<br>• pH strongly influenced structure: at pH 6 → strong 3D gel network (porous, aggregated microstructure); at pH 7–8 → transition toward colloidal precipitate due to negatively charged silanol groups.<br>• Yield stress and viscosity decreased with increasing pH (from 3.0 Pa and 100 mPa·s at pH 6 → 0.77 Pa and 15 mPa·s at pH 8).<br>• Interaction forces reversed with pH: attractive (gel formation) at pH 6, repulsive (precipitation) at pH 7–8.<br>• Gelation delayed at higher frequencies, G′/G″ crossover times increased from 3 min (0.1 Hz) to 8 min (0.5 Hz).<br>• Concluded that rheological behavior depends on balance between van der Waals attraction and electrostatic repulsion, defining gel strength and thixotropy.

Gorrepati, E.A., Wongthahan, P., Raha, S., Fogler, H.S. (2010) (Gorrepati et al. 2010)	Monosilicic acid (from $Na_2SiO_3 \cdot 9H_2O$) acidified with HCl (2–8 M); additional salts (1 M $AlCl_3$, $CaCl_2$, $MgCl_2$, NaCl, CsCl).	Pure solution; dissolution experiments.	• Identified a two-step polymerization mechanism in acidic media: (1) rapid dimerization of monosilicic acid to form ~5 nm primary particles, followed by (2) flocculation and exponential growth of aggregates up to 200 nm.<br>• Polymerization and flocculation rates increase exponentially with HCl concentration (2–8 M).<br>• Salts accelerate polymerization and flocculation in order: $AlCl_3$ > $CaCl_2$ > $MgCl_2$ > NaCl > CsCl > no salt.<br>• Ionic strength dominates cation type; higher ionic strength increases aggregation rate constants and collision efficiency.<br>• At high ionic strength, traditional DLVO theory fails, suggested short-range hydration or steric (silanol "hair") forces influence aggregation.
Mollamahmutoğlu, M., Avcı, E., Deveci, E., Yıldırım, E. (2021) (Mollamahmutoğlu et al. 2021)	Sodium silicate–sodium dihydrogen phosphate, first use of phosphate as inorganic, non-toxic hardener.	River sand (Karacabey, Turkey), fine, medium, and mixed gradations; five types. Relative density 30%.	• Introduced NaH_2PO_4 as a novel silicate hardener, yielding stable, low-viscosity grouts.<br>• Gel time prolonged and viscosity increased with higher phosphate content.<br>• Syneresis increased with phosphate content but slowed after 18 days.<br>• Air-dried UCS: 740–1529 kPa (increases with curing time and phosphate dose). Wet-cured UCS: 49–293 kPa (decreases with time due to syneresis). Air-dried samples up to 11.8 times stronger than wet-cured.<br>• Permeability reduced 1–5 orders of magnitude.<br>• Strength loss under wet curing attributed to water release (syneresis) reducing gel volume and inter-particle bonding.<br>• Fine sand improved grout retention and strength.<br>• Demonstrated strong phosphate–silicate network formation providing enhanced mechanical stability and reduced permeability.
Salehzadeh, H., Hassanlourad, M., Shahnazari, H. (2012) (Salehzadeh et al. 2012)	Sodium silicate with formamide ($HCONH_2$) as reactant and sodium aluminate ($NaAlO_2$) as accelerator.	Three carbonate sands, Kish, Hormoz (Persian Gulf), and Rock.	• Sodium silicate grout bonded grains effectively, enhancing shear strength depending on grout concentration and confining pressure.<br>• Stronger grout increased cohesion and delayed bond failure; weaker grout behaved similarly to ungrouted sand beyond ~14% strain.<br>• Particle shape and grain crushing influenced bond efficiency.<br>• Over consolidation (OCR = 8) increased yield strength and changed volumetric response from contractive to dilative.<br>• Developed a new stress–dilation relationship for grouted sands including bond and confining pressure terms.
Avci, E., Mollamahmutoğlu, M., Deveci, E. (2022) (Avci et al. 2022)	Sodium silicate with formamide as hardener.	Silt and silty fine sand (Kızılırmak River, Turkey), five gradations (0–100% silt); relative density 30%.	• Successfully grouted both silt and silty sand, demonstrating that formamide–silicate systems can penetrate fine-grained soils.<br>• Higher silicate and formamide content shortened gel time and increased viscosity and syneresis.<br>• Air-dried specimens gained strength with time (up to 6.9 MPa), while wet-cured specimens lost strength (up to 56% decrease by 90 days) due to syneresis-driven water loss and bond weakening.<br>• Strength of higher silicate is 3.7 times higher than lower silicate grouts.<br>• Permeability reduced by 2–4 orders of magnitude (to 10^{-6}–10^{-5} cm/s); slightly increased with time due to syneresis and gel shrinkage.
Mollamahmutoğlu, M., & Avci, E. (2020) (Mollamahmutoğlu and Avci 2020)	Sodium silicate with formamide as the organic hardener.	River sand (Kızılırmak, Turkey), five gradations (fine to medium), 30%, 50%, and 70% relative density.	• Gel time decreased and viscosity increased with higher silicate content.<br>• Syneresis increased with silicate content up to 50%, then declined beyond that due to gel densification.<br>• Penetrability decreased with higher silicate content, finer sand, and higher relative density; required higher injection pressures (9–50 kPa).<br>• Strength (UC & UU) rose with silicate content and fine fraction; optimum at 60% fine + 40% medium sand.<br>• Air-dried samples were ~3.6 times stronger (0.48–2.52 MPa) than wet-cured ones (0.11–0.80 MPa) due to dehydration-induced matrix tightening.<br>• Syneresis weakened wet-cured samples by degrading gel bonding and reducing cohesion over time.<br>• Air-dried strength increased up to day 56 and stabilized; wet-cured strength decreased with time.

U.S. Army Corps of Engineers (1995) (US Army Corps of Engineers 1995)	phosphoric acid, CO_2, $CaCl_2$, $MgSO_4$, $Al_2(SO_4)_3$, formamide, glyoxal, and acetic ester.	Granular soils and fractured rock.	• Comprehensive guidance on grout design, mixing, and injection for field practice.<br>• Strength and permeability depend on silicate concentration, soil grain size, and curing conditions; loose materials gain strength primarily from gel matrix.<br>• Durability: ≥ 35 % silicate → resistant to freezing/thawing and wet/dry cycles; < 30 % → temporary use only.<br>• Environmental note: Silicate systems are among the least toxic and most compatible grouts.
Xu, Y.; Wei, T.; Chen, G.; Ma, J.; Yan, M. (2023) (Xu et al. 2023)	Sodium silicate (water glass) modified with NaOH (no external hardener).	River sand (Chengdu, China), well-graded.	• Investigated temperature and curing-path effects on sodium silicate–solidified sand (20–100 °C).<br>• Defined two phases: strength-rising and strength-deterioration stages.<br>• UCS increased with Baume degree: up to 9.05 MPa (30° Bé) at room temperature; 18.8 MPa after 3 days RT + 4 days high-temperature curing.<br>• High temperature (100 °C) accelerated dehydration/polymerization, increasing strength, but also accelerated late-stage deterioration in low Baume gels.<br>• SEM revealed progressive transformation of gels: from underhydrated gel to room-temperature rigid gel to high-temperature rigid gel, with formation of acicular and columnar SiO_2 crystals.<br>• High Baume gels developed denser microstructures and slower degradation, while low Baume gels showed faster dehydration and cracking.<br>• Proposed a strength evolution mechanism: early rapid polymerization, mid-term densification, late dehydration-induced cracking.
Porcino, D.; Marcianò, V.; Granata, R. (2012) (Porcino et al. 2012)	Sodium silicate with inorganic hardener.	Ticino silica sand (medium, uniform; D_{50} = 0.6 mm; quartz 30%, feldspar 65%)	• Grouting formed weak cementation bonds between sand grains, leading to lightly cemented sand (UCS < 100 kPa).<br>• Chemical grouting significantly reduced permeability (1.2×10^{-7}–2.3×10^{-7} cm/s).<br>• Shear stiffness and shear-wave velocity increased due to partial cementation and bond formation.<br>• Treated sand showed brittle response and enhanced dilatancy during drained triaxial shear.<br>• Grouted sand demonstrated delayed pore pressure buildup and slower stiffness degradation under cyclic loading.<br>• Syneresis was negligible due to the presence of medium-fine sand, which confined the gel and retained water.
Chen, Xu, Zhang & Yan (2024) (Chen et al. 2023)	Sodium silicate modified with NaOH (to adjust modulus 1.5–3.0) and water (Baume 15–30).	River sand from Chengdu (China), $D_{50} \approx$ 0.3–0.6 mm; used as representative sandy soil.	• Consolidation rate: Shorter at high modulus, slower at high Baume. High Baume (denser solution) forms more gel but cures slowly due to limited dehydration.<br>• Microstructure: SEM and NMR showed gel coats sand particles, filling pores and binding grains. High Baume led to thick gel layers and large agglomerates with bigger pores; low Baume formed thin, compact coatings with fine pores.<br>• Disintegration behaviour: High Baume specimens disintegrated rapidly under water immersion (up to 95% loss), while low Baume samples remained stable (<10% loss). Poor water stability was linked to incompletely dehydrated gel layers that dissolve when immersed.<br>• Mechanism: Two key processes, (1) gel precipitation and (2) dehydration/condensation. Under strong alkalinity, Na^+ ions attack SiO_2 on sand surfaces forming monomeric silicates (HO–Si(ONa)$_3$, HO–Al(ONa)$_3$), which condense to form Si–O–Si and Si–O–Al linkages, coating sand particles and enhancing bonding.<br>• Mechanical response: Higher Baume produced stronger but more brittle specimens.<br>• Implications: High Baume solutions yield high strength but poor water resistance; low Baume solutions form more stable gels suitable for wet conditions.

Guo, Zhang, Bi & Zhang (2024) (Guo et al. 2024)	Sodium silicate (water glass) — used directly (no added hardener) at various Baume degrees (42°, 47°, 52°).	Loess soil from Xi' an, China (fine silt-dominated, porous, weakly cemented)	• UCS increased with higher Na_2SiO_3 content, higher Baume degree, and longer curing; maximum strength at 20% and 52° Be after 28 days.<br>• Chemical reaction: Na_2SiO_3 interacts with native Ca^{2+} to form Ca-silicate hydrates, binding particles and filling pores.<br>• Microstructure (SEM/XRD/MIP): progressive filling of macropores and formation of dense gel films around soil grains; porosity decreases and particle size coarsens with curing.<br>• Three pore-size peaks (nano–micro–macro) evolve into finer, denser structures; mesopores dominate after 14 days.<br>• Failure modes: transition from plastic to brittle shear with increasing content and curing; stronger samples showed brittle fracture due to cementation.<br>• Empirical model fitted UCS as a function of Baume degree, curing time, and silicate content ($R^2 > 0.9$).<br>• Reinforcement mechanism: pore filling + particle bonding through silica gel and Ca–silicate crystallization → compact, cohesive matrix.
Wang, X.; Wang, C.; Li, P.; Tian, D.; Wang, J.; Liu, B. (2023) (Wang et al. 2023)	Acidic sodium silicate grout using phosphoric acid (H_3PO_4) as the hardener.	Poorly graded medium sand.	• Silica gel formation and growth during curing were the main causes of strength increase; silica content rose from 60 % (1 d) → 70 % (3 d) → 80 % (28 d).<br>• SEM/XRD analyses: revealed progressive pore filling and bonding between sand grains by silica gel, forming continuous Si–O–Si networks.<br>• High water content reduced bonding and syneresis, leading to lower UCS due to retained free water at gel–sand interfaces.<br>• Identified optimal Baume (30 °Be) and pH (3.4–3.8) for balanced strength and injectability.<br>• Field tests in a tunnel sand stratum confirmed stability and diffusion radius of 26–38 cm with UCS = 0.2–0.25 MPa.<br>• Proposed mechanism: phosphoric acid lowers pH, triggers polymerization and Si–O–Si condensation forming dense, acid-resistant silica networks within sand pores.
Xu, Y., Zhang, Y., Huang, J., Chen, G. (2022) (Xu et al. 2022)	Sodium silicate modified with NaOH and water (no external hardener).	Fine sand from Chengdu River, China.	• Strength of water-glass-cured sand exhibits a saturation value controlled by Baume degree: low-Baume grouts cured faster but reached lower peak strengths (<3 MPa), while high-Baume grouts cured slower but reached higher strengths (>8 MPa).<br>• SEM and NMR analyses revealed progressive gel condensation, water migration, and silica polymerization forming Si–O–Si networks coating sand grains.<br>• Gel dehydration and condensation reduced pore size and increased cohesion.<br>• Two coupled processes, water-glass migration and progressive curing, govern consolidation: low-Baume gels migrate faster and cure near the surface, producing sandy cores; high-Baume gels migrate slower and solidify uniformly.<br>• Mechanism: Na^+ attack on SiO_2 and Al-silicates releases monomers (HO–Si$(ONa)_3$, HO–Al$(ONa)_3$) that polymerize into Si–O–Si/Si–O–Al gels coating sand grains, enhancing bonding and mechanical integrity.
Mollamahmutoglu, M.; Littlejohn, G.S. (1995) (Mollamahmutoğlu M. and Littlejohn G 1995)	(1) Geoseal MQ-5 (commercial silicate grout), (2) Sodium silicate + Hardener 600B (ester type).	Leighton Buzzard Sand and Thames Ballast Sand	• Compared two chemical grout systems (Geoseal MQ-5 and Silicate-600B) in terms of viscosity, syneresis, gelation time, and groutability.<br>• Viscosity–time relationships showed steady increase with hardener dosage; optimum range for permeation ≤ 15 cP.<br>• Syneresis: increased with silicate content but reduced with higher neutralization; nearly absent when grouted into sand.<br>• Permeation tests: both grouts successfully penetrated medium to fine sands, achieving uniform gel distribution.

Cui, Y.; Tan, Z.; Han, D.; Song, J. (2022) (Cui et al. 2022)	Sodium silicate (40°Bé, modulus 3.3) with diacid ester curing agent.	Poorly graded silty fine sand.	• Developed a new diacid-ester–based sodium silicate grout (DS grout) for water-rich silty fine sands.<br>• Gel time: adjustable between 3 s–9 min depending on catalyst concentration; shortest gel time at optimal H^+ concentration.<br>• Strength: compressive strength of grouted sand = 1.6–3.0 MPa (increased with silicate concentration and ester dosage).<br>• Durability: DS grout retained >60% strength after 28 days in air and water, outperforming phosphate–silicate grout (which collapsed in air).<br>• Microscopy (SEM/IR): confirmed polymerization of Si–O–Si and formation of carboxylate bonds; denser gel matrix than acid-modified silicate.<br>• Model tests: diffusion radius ≈9.5 cm, compressive strength 2.0–2.15 MPa (≈4 times stronger than phosphate–silicate).<br>• Field application: in tunnel excavation through water-rich sand in Beijing — DS grout achieved stable, impermeable reinforcement (UCS ≈ 2 MPa) at 0.5 MPa injection pressure, outperforming phosphate–silicate (0.21 MPa at 2 MPa pressure).
Mollamahmutoğlu, M.; Avci, E.; Tomaç, S.K.; Köse, D.A. (2017) (Mollamahmutoğlu et al. 2017)	Sodium silicate–boric acid grout	Kızılırmak River sand, 5 gradations (fine to medium mixtures) at 30–70% relative densities	• Introduced boric acid as a novel neutralizing reactant forming polymeric sodium borosilicate gel through esterification.<br>• Gel time: increased with higher silicate content (35–62 min at 20 °C); decreased with higher boric acid concentration.<br>• Viscosity: rose with both silicate and boric acid content (3.6–4.3 cP).<br>• Syneresis: severe for neat gels (up to 87% after 150 days), but less pronounced in grouted sands due to pore confinement; higher silicate and boric acid content both increased syneresis.<br>• Strength: wet-cured UCS = 0.12–0.27 MPa; air-dried UCS = 0.22–0.44 MPa; air-dried specimens ≈ 1.7 times stronger than wet-cured. Strength peaked at 14–28 days then declined due to syneresis-induced shrinkage.<br>• Permeability: decreased by 2–3 orders of magnitude (10^{-5}–10^{-6} cm/s); reduced further with higher boric acid but increased slightly with more silicate content.<br>• Durability: strength and impermeability deteriorated slowly with time because of water loss from the gel matrix.
Wilhelm & Kind (2014) (Wilhelm and Kind 2014)	Sodium silicate ($Na_2O{\cdot}3.3SiO_2$, 40 wt%) acidified with sulfuric acid (H_2SO_4, 50 wt%)	Pure gel (no soil medium)	• Investigated natural and enforced syneresis in precipitated silica gels prepared by mixing sodium silicate and sulfuric acid under controlled pH and temperature.<br>• Developed a predictive model linking natural ($\Delta p = 0$) and enforced ($\Delta p > 0$) syneresis using empirical equations for maximum volume decrease ($\Delta V/V_0$) and characteristic time constant (τ).<br>• Found that natural syneresis shows ~20% volume shrinkage ($\Delta V/V_0 \approx 0.2$) at 20–60 °C, while enforced syneresis ($\Delta p = 15.5$ bar) reached 87% shrinkage within hours.<br>• Increasing temperature (20→60 °C) accelerates the rate of syneresis but does not significantly affect total shrinkage.<br>• Higher external pressure yields faster and greater shrinkage, mimicking long-term aging effects.<br>• Demonstrated that enforced syneresis can predict natural syneresis behavior, reducing analysis time from days to hours.<br>• Proposed potential modification of gels (e.g., replacing hydroxyl groups with methylene groups) to stabilize silica against post-gelation shrinkage.
Wilhelm & Kind (2015) (Wilhelm and Kind 2015)	Sodium silicate + sulfuric acid	Pure precipitated silica gel	• Investigated natural vs enforced syneresis under varying pH (0–10), temperature (20–60 °C), and sample size (3–12 mm).<br>• Natural syneresis: Acid-catalyzed gels showed constant shrinkage ($\Delta V/V_0 \approx 0.20$) independent of temperature; base-catalyzed gels showed temperature-dependent shrinkage (0.30–0.51).<br>• Shrinkage and consolidation rate increased with temperature and smaller sample size.<br>• Enforced syneresis: External pressure (0.2–7 bar) accelerated consolidation, with shrinkage up to 0.9 at 7 bar; analogous behavior to natural syneresis observed.<br>• Developed a correlative model linking enforced and natural syneresis.

Tognonvi, Rossignol & Bonnet (2011) (Tognonvi et al. 2011)	Sodium silicate solution (Si/Na = 1.71, [Si] = 7 mol/L, pH = 11.56) acidified with HCl	Pure solution and resulting gels (no soil medium)	• Studied the gelation and ripening behavior of sodium silicate under controlled pH (9–11.56) and concentration.<br>• Gelation time increases with pH and decreases with silicon concentration; chlorine ions act only as spectator ions.<br>• Syneresis and ripening mechanisms involve dissolution–precipitation and pore coalescence, leading to consolidation of gels.<br>• Demonstrated strong dependence of gel structure and stability on pH and silicate concentration, linking them directly to syneresis rate and aging behavior.
Tognonvi, Lecomte, Rossignol & Bonnet (2019) (Tognonvi et al. 2019)	Sodium silicate ($Na_2O{\cdot}3.41SiO_2$) solution partially acidified with HCl (0.5–2 M)	Pure gel system; aging/ripening at room temperature for 30–150 days	• Investigated acidification-induced gelation and ripening of sodium silicate in mildly basic media.<br>• Identified four gel types (A–D): (A) clear stable solution; (B) reversible physical gels (Van der Waals/H-bonded); (C) soluble white gels undergoing unidirectional shrinkage; (D) irreversible white gels with strong syneresis and silica precipitation.<br>• syneresis driven by dissolution–precipitation and condensation reactions.<br>• Syneresis and gel stability governed by pH and Si concentration: lowering pH to 9–10.75 led to strong shrinkage and phase separation into Na-rich soluble phase ($NaSi_{1.87}O_{4.24}$) and silica-rich insoluble phase ($NaSi_{12.66}O_{25.82}$).<br>• Physical gels stable for months; chemical (irreversible) gels consolidated into dense, low-surface-area solids.<br>• Concluded that gel shrinkage and structure are controlled by charge balance of silicate species and acid-induced neutralization processes.
Avcı (2017) (Avci 2017)	Sodium silicate blended with glyoxal (organic acid derivative) as hardener	Quartz sand from Kızılırmak River (fine to medium), compacted at 30%, 50%, and 70% relative densities	• Gel time decreased with increasing sodium silicate content (from 157 to 62 min at 20 °C).<br>• Viscosity increased with silicate concentration (1.95 → 4.89 cP).<br>• Syneresis increased with silicate content up to 37%, then decreased beyond that (max 80%).<br>• Permeability of grouted sands decreased by 2–5 orders of magnitude compared to ungrouted samples (from ~10^{-3}–10^{-1} cm/s to 10^{-6} cm/s range).<br>• Permeability increased with time due to syneresis (matrix shrinkage).<br>• Finer sands and higher relative densities produced lower permeability and higher injection pressures.<br>• Demonstrated that glyoxal–sodium silicate grouts are effective in reducing permeability but subject to gradual degradation from syneresis over 150 days.
Hamouda & Amiri (2014) (Hamouda and Amiri 2014)	sodium silicate solution; gelation controlled by acid titration (HCl) and divalent ions (Ca^{2+}, Mg^{2+})	Pure gels in synthetic seawater (SSW), low-salinity water (LSW), and distilled water (DW)	• Studied combined effects of pH, temperature, salinity, shear rate, and divalent ions on gelation kinetics, gel strength, and syneresis.<br>• Lower pH and higher silicate concentration accelerate gelation.<br>• Divalent ions (Ca^{2+}, Mg^{2+}) shorten gelation time and increase gel strength up to threefold; Mg^{2+} slightly less effective.<br>• At 0.009 M divalent ions, gel strength tripled and was reached 8 times faster than ion-free solution.<br>• Temperature: increased polymerization rate but reduced gel strength by 39% at 50 °C; strength partially recovered at 80 °C due to increased solubility and pH reduction.<br>• Shear (1000 s^{-1}) before gelation accelerated setting and increased strength; shear after gelation reduced viscosity temporarily but allowed reformation.<br>• Syneresis increased with temperature and divalent ion content; Ca^{2+} enhanced shrinkage ~3 times more than Mg^{2+}.<br>• Proposed predictive model (empirical charts) combining pH, temperature, and ion concentration to estimate gelation time and strength.

May et al. (1986) (James H. May et al. 1986)	Sodium silicate (30% and 50%), acrylate, urethane, and portland cement	Medium and fine sand, exposed to 12 simulated hazardous waste liquids (acids, bases, salts, and organics)	• Evaluated grout chemical compatibility and sealing efficiency under aggressive waste conditions.<br>• Strong bases (NaOH, NH_4OH) inhibited gelation or caused partial dissolution of silicate gels.<br>• Acidic or metallic salts (HCl, H_2SO_4, $CuSO_4$, NH_4Cl) caused instantaneous gelation ("flash set") and formation of insoluble silicate precipitates.<br>• Organic solvents and fuels (benzene, toluene, oil) induced swelling, softening, and detachment from soil particles, increasing permeability.<br>• After ~20 days, silicate and acrylate grouts remained largely intact, while urethane degraded completely; syneresis observed in all chemical grouts under saline or alkaline conditions.<br>• Concluded that silicate grouts exhibit moderate chemical durability, but their performance strongly depends on groundwater chemistry, particularly pH, ionic strength, and contaminant composition.<br>• Recommended using blended systems (e.g., silicate–cement or silicate–bentonite) to improve long-term stability in contaminated sites.
Krizek & Spino (2000) (Krizek and Spino 2000)	Sodium silicate (50%) with ethyl acetate and formamide as hardeners	Maryland sand (poorly graded, quartz-rich, slightly cemented, medium to fine sand)	• Field injection into natural sand at 3.6 m depth; specimens cured 17–24 months before testing.<br>• Syneresis observed during curing; permeability of grouted sand ~10^{-4} cm/s, two orders of magnitude lower than ungrouted sand.<br>• No clear variation in strength, modulus, or permeability with distance (up to 1.7 m) from injection point.<br>• Shear strength increased cohesion from ~40 to 1000–1500 kN/m², friction angle remained ~28°.<br>• Dissolution with NaOH (1.5–2 N) allowed quantification of grout content.<br>• Coring slightly reduced measured modulus and strength (~10–25%) compared to hand-trimmed specimens.<br>• Concluded that silicate grout forms a fairly homogeneous, durable mass with minor anisotropy and stable long-term mechanical properties.
Bodocsi & Bowers (1991) (Bodocsi and Bowers 1991)	Sodium silicate (60%) + formamide (10%) + $CaCl_2$ (5%) + water (25%); compared with glyoxal-modified and sodium aluminate-modified silicate grouts	Natural medium mason' s sand	• Evaluated long-term permeability and chemical durability of various grouts under exposure to 10 industrial chemicals and 2 real-site wastes (paint and refinery effluents).<br>• Unmodified sodium silicate grout: baseline permeability ~5×10^{-5} cm/s (too high for containment), but showed little change in permeability with most chemicals; slight increase with acetone; permeability decreased with refinery waste and xylene due to microbial growth plugging pores.<br>• Glyoxal-modified silicate grout: permeability ~1.9×10^{-5} cm/s; resistant to $CuSO_4$ and NaOH, minor deterioration with xylene.<br>• Microbial interactions observed: biological growth in refinery waste reduced permeability by up to four orders of magnitude—implying potential for bio-clogging in contaminated environments.
Mollamahmutoğlu & Avcı (2016) (Mollamahmutoğlu and Avci 2016)	Sodium silicate grouts with formamide as organic hardener	Quartz sand (fine and medium fractions from Kızılırmak River, Turkey), relative densities: 30%, 50%, 70%	• Investigated syneresis and its influence on permeability of grouted sand for 720 days (20°C).<br>• Gel time decreased from 425 to 185 min as silicate content increased (30 → 70%).<br>• Viscosity increased with silicate content (1.67 → 4.28 cP).<br>• Syneresis increased up to 50% silicate (max 36.5%), then decreased for higher concentrations.<br>• Permeability reduced by 1–4 orders of magnitude after grouting (10^{-3}–10^{-1} → 10^{-5} cm/s), but increased ~9% over 150 days due to syneresis-driven shrinkage.<br>• Grouting pressure rose with increasing silicate content and soil density (0.04 → 0.70 MPa).<br>• Concluded that syneresis effects are minor in sand–gel systems compared to pure gels, but long-term shrinkage remains relevant for groundwater sealing applications.

Gonzalez & Vipulanandan (2007) (Gonzalez and Vipulanandan 2007)	Sodium silicate ($Na_2SiO_3{\cdot}3H_2O$) with dimethyl ester (DME) as organic hardener	Medium dense sand	• Gel time decreased linearly with DME content (60 → 37 min at 23°C).<br>• Syneresis in neat grout: 24–36%; in grouted sand: only 4.6%. ~70% of syneresis occurred within 24 h.<br>• Optimum DME = 7%, yielding the highest compressive strength (1.5 MPa after 14 days).<br>• Proposed empirical relationships linking stress level and time-to-failure; confirmed that creep failure is composition-dependent.<br>• Established correlation between DME concentration, gel time, strength, and creep stability.<br>• Concluded that higher organic reactant concentration improves early strength and creep resistance but may increase syneresis.
Ata & Vipulanandan (1998) (Ata and Vipulanandan 1998)	Sodium silicate ($Na_2SiO_3{\cdot}3H_2O$) grout with organic reagent mixture (50% ethyl acetate + 50% formamide) as hardener	Ottawa 20–30 sand and limestone aggregates; cured up to 2 years	• Investigated cohesive strength (grout) and adhesive bonding strength (grout–sand interface) over 730 days.<br>• Grout strength doubled between 3 and 14 days (345 → 690 kPa) and increased by 36% at 28 days; reached ~1 MPa after 2 years.<br>• Adhesive strength between silicate grout and quartz: ~275–520 kPa (plateau after 7 days); for limestone: ~1,000 kPa after 1 year (due to chemical reaction with $CaCO_3$).<br>• Unconfined compressive strength (UCS) of grouted sand: 0.55 MPa (3 d) → 0.62 MPa (28 d), stable after 2 years.<br>• Developed hyperbolic relationship linking grout cohesive and adhesive strengths to grouted-sand compressive strength.<br>• Concluded that strength is limited by the weaker of grout cohesion or adhesion, and proposed design curves relating sand porosity, grout strength, and adhesive bonding for predicting field performance.
Delfosse-Ribay et al. (2006) (Delfosse-Ribay et al. 2006)	Sodium silicate grout with organic hardener (8%), compared with microfine cement and mineral grouts	Fontainebleau sand, grouted columns; tests included unconfined and triaxial confined creep	• Compared chemically unstable silicate grout with chemically stable microfine cement and mineral grouts.<br>• Silicate grout was subject to syneresis and chemical degradation, releasing soda and organics; banned in some EU countries due to environmental risks.<br>• Confining pressure (100 kPa) reduced creep slope by ~6.5 times for silicate grout; similar stabilizing effects observed for mineral grout.<br>• Sand preparation affected results, manually compacted sand exhibited ~40 times higher creep strain than pluviated sand (due to lower homogeneity).<br>• Proposed method for predicting creep behaviour of grouted sand from pure sand and pure grout behaviour, implying creep strain of grout dominates.
Littlejohn & Mollamahmutoğlu (1994) (Littlejohn and Mollamahmutoğlu 1994)	Sodium silicate with ester hardener (Rhone-Poulenc 600B)	Leighton Buzzard sand, medium dense, grouted and cured at 100% RH	• UCS increased with silicate content: 180–1,400 kPa after 24 h; strength stabilized after 1 day and remained constant for 1 year.<br>• Creep tests (unconfined and confined) at stress ratios 0.15–0.8 showed that failure time decreased with increasing silicate content and applied stress.<br>• Under unconfined conditions, failure occurred within 8 min to 7 days depending on stress level.<br>• At 100 kPa confinement, low-silicate (40–50%) mixes stable up to 0.5 stress ratio; higher-silicate (60–80%) failed even at 0.3 ratio.<br>• At 300 kPa confinement, all silicate contents (40–80%) stabilized (no failure at 0.3–0.8 stress ratio).<br>• Demonstrated that silicate-ester grouted sand exhibits viscoelastic (hydrogel-like) time-dependent deformation.

Vipulanandan & Krizek (1986) (Vipulanandan and Krizek 1986)	Sodium silicate with ethyl acetate + formamide (1:1) as organic hardeners	Ottawa 20–30 sand, cured at 20 °C	• Developed a mechanistic model for chemically grouted sand as a two-phase composite governed by grout cohesion, sand–grout adhesion, and particle interaction.<br>• Pure grout tests: linear tensile and compressive behaviour; modulus increased with curing (up to ~14 days).<br>• Adhesion tests (tension, shear, mixed mode): adhesive tensile and shear strength peaked at 7–10 days, then decreased due to shrinkage and microcracking.<br>• Grouted sand: tensile and compressive strength increased with curing.<br>• Failure characterized by mixed cohesive and adhesive debonding, governed by weakest link.<br>• Proposed tensile strength and stiffness models that predict grouted sand properties from grout and interface parameters.<br>• Identified matrix cracking near sand–grout interfaces as dominant failure mode.<br>• Curing improved modulus and brittleness; long-term stability limited by interface degradation and shrinkage-induced stress.
Kaga & Yonekura (1991) (Kaga and Yonekura 1991)	Sodium silicate with organic hardener	Five types of natural sands (fine to coarse) with different densities (Dr = 0.16–0.95) and specific surface areas (50–180 cm^2/g).	• Developed a universal exponential model linking grouted sand strength and pure grout strength, correlating experimental and theoretical results with >97% accuracy.<br>• Grouted sand strength proportional to density (Dr) and pure grout strength within specific limits; beyond these limits, proportionality breaks down.<br>• Applied Mohr–Coulomb failure criterion and a two-phase composite model to explain strength mechanisms, showing that cohesion gain depends on grout tensile strength, porosity, and surface area.<br>• Derived analytical equation linking unconfined strength to sand porosity and grout tensile strength.<br>• Found that fine particles (<74 μm) up to 5% do not influence overall strength, as they do not alter the load-bearing skeleton.<br>• Demonstrated that sand surface area is the dominant factor controlling strength prediction accuracy; ignoring very fine particles improves correlation.<br>• Provided empirical relationships for model coefficients as functions of density, porosity, and surface area.
Han & Tan (2011) (Han and Tan 2012)	Sodium silicate with H_2SO_4 as hardener	Various H_2SO_4:Na_2SiO_3 ratios (1:1–1:4.5)	• Investigated influence of groundwater pH and ionic species on the gelling time and durability of chemical grouts.<br>• Gelling time of sodium silicate varied nonlinearly with pH, maximum (~390 min) at pH ≈ 1.5; both more acidic and more alkaline conditions shortened gelling time.<br>• Acidic or alkaline groundwater affects both gelation kinetics and long-term durability due to erosion or decomposition of gel.<br>• Dissolved ions such as Ca^{2+} and CO_3^{2-} can react with grout to form precipitates ($CaCO_3$), reducing permeability and limiting penetration of the grout.<br>• Emphasized that site-specific groundwater chemistry (pH, CO_2 partial pressure, ion type) should be analysed before grouting design.
Vipulanandan & Ata (2000) (Vipulanandan and Ata 2000)	Sodium silicate with formamide and ethyl acetate as organic hardener	Dry Ottawa 20–30 sand	• Investigated dynamic, cyclic, and fatigue properties of silicate-grouted sand.<br>• Grouted sand failed below 0.3% axial strain; deformation evolved in three stages (initial, steady, failure).<br>• Secant modulus decreased linearly up to ≈ 80% of life cycle; damping ratio increased from ~3% to ~6% before failure.<br>• Damping ratio decreased with curing, stabilizing after 90 days (~3–4% for mature gels).<br>• Demonstrated that silicate-grouted sands exhibit viscous-type damping, and dynamic modulus and fatigue behaviour can be quantitatively predicted.

Mollamahmutoglu & Littlejohn (1997) (Mollamahmutoglu and Littlejohn 1997)	Sodium silicate (SiO_2:Na_2O = 3) with Rhone Poulenc 600B ester hardener	Leighton Buzzard sand, grouted by permeation under 20 kPa injection pressure	• Investigated temperature sensitivity of creep behaviour in silicate–600B ester–grouted sand.<br>• Triaxial creep tests under confining pressure of 100 kPa and stress ratios of 0.2–0.6; temperature varied cyclically between 20°C and 30°C.<br>• Creep strain increased by 1–15% when temperature rose from 20°C to 30°C for 40–60% silicate mixes; negligible effect for 70–80% mixes.<br>• Creep rate increased 1–3% for low-silicate mixes during heating; reversed during cooling.<br>• Low-silicate gels (40–60%) exhibited high syneresis → pore water pressure rise → reduction in effective stress → accelerated creep.<br>• High-silicate gels (≥70%) displayed stable creep and temperature-insensitive performance due to higher gel stiffness and lower syneresis.<br>• Concluded that ambient temperature fluctuations can substantially affect deformation behaviour and long-term stability of chemically grouted sands.
Komine (2000) (Komine 2000)	Hydrated sodium silicate (35%) with glyoxal (50 mL) and phosphoric acid (12 mL) as hardeners	Mikawa silica sands	• Developed a non-destructive evaluation method using electrical resistivity tomography (ERT) to delineate chemically grouted zones.<br>• Grouted using permeation and fracture–permeation modes.<br>• Electrical resistivity of silicate gel ≈ 0.62 Ω·m, significantly lower than pore water (27–48 Ω·m) or sand matrix (~560 Ω·m).<br>• Resistivity tomography accurately mapped the spatial extent of the improved (grouted) region, correlating with destructive verification.<br>• Derived an empirical model linking resistivity ratio to grout:void ratio (α) to estimate grouting effectiveness quantitatively.<br>• Identified critical grout:void ratios for effective solidification: >85% (coarse sand), >60% (medium sand), >40% (fine sand).<br>• Measured unconfined compressive strength of 148–369 kPa and permeability 1.6×10^{-8}–2.9×10^{-9} m/s, matching design criteria.<br>• Concluded that ERT provides a reliable tool for real-time monitoring and post-verification of chemical grouting performance.
Komine (1997) (Komine 1997)	Hydrated sodium silicate with glyoxal + phosphoric acid as hardeners	Toyoura sand, Mikawa silicate sands, saturated and grouted by permeation	• Investigated how electrical resistivity can be used to evaluate the quality and extent of silicate grouting.<br>• Found that resistivity of grouted sand decreases sharply with increasing grout/void ratio (α), stabilizing below ~ 5 Ω·m when α > 60%.<br>• Grout gel continuity controls resistivity; continuity achieved at α ≈ 60 % for Toyoura sand.<br>• Large-grain sands have higher resistivity than fine sands at equal α because grout continuity breaks more easily.<br>• Electrical resistivity of saturated sand correlates strongly with that of pore water but not with grain size.<br>• Developed analytical models (series + parallel) for predicting resistivity of saturated and grouted sands and used them to derive grout/void ratio from resistivity data.<br>• Validated models experimentally, results agreed with calculated α–ρ relationships within ±10 %.<br>• Proposed that resistivity tomography can non-destructively assess grouted region continuity and quality in field applications.
Pham Huy Giao, Nguyen Quoc Cuong, & Loke Meng Heng (2012) (Pham et al. 2011)	Sodium silicate with formamide as reactant	Compacted sandy soil; grouting in 30×30×60 cm tank; monitored via ERT	• Demonstrated laboratory-scale monitoring of chemical permeation grouting using ERT.<br>• Grout resistivity initially 0.3 Ω·m, increasing to 0.55 Ω·m after 15 hours, correlating with gelation and hardening.<br>• Base sand resistivity ≈ 100 Ω·m; hence clear resistivity contrast (≈200×) allowed accurate mapping of grout distribution.<br>• Cross-borehole bipole-bipole array provided best imaging resolution, detecting grout front and its gravity-driven migration downward over 12 hours.<br>• Validated through synthetic modelling and inversion (using RES3DMOD & RES3DINV).<br>• Concluded that ERT effectively captures the temporal evolution and geometry of the grouted region; suitable for real-time field monitoring in subsurface conditions.

M. Mollamahmutoglu (1999) (Mollamahmutoglu 1999)	Sodium silicate with Hardener 600B	Leighton Buzzard sand	• Investigated multi-stage creep behaviour of chemically grouted sand under unconfined and confined (100 kPa) conditions.<br>• Creep strain increased with higher stress ratio and silicate content; higher stresses led to accelerated strain rate and eventual creep rupture.<br>• Increasing silicate content from 40% to 50% shortened creep duration, 3000 min → 100 min for same stress ratio (0.4).<br>• Confining pressure (100 kPa) reduced creep rate and delayed rupture (stabilized after 10,000 min).<br>• Highlighted the time-dependent deformation risks in grouted soils subjected to sustained loading.
Littlejohn, G.; Concannon, M.; Wright, R. (1997) (Littlejohn et al. 1997)	Sodium silicate with R100 ester hardener	Leighton Buzzard sand	• Comprehensive study of viscosity, setting time, syneresis, and compressive strength of silicate–R100 ester grouts.<br>• Initial viscosities: 2–30 cP; setting time decreased with higher hardener and temperature.<br>• Syneresis: increased with silicate content up to 60%, decreased with higher neutralization; negligible in sand.<br>• Neutralization control critical for durability, higher degree improves strength but excessive neutralization can cause premature setting.<br>• Temperature sensitivity: setting time halved between 10–30 °C; alkaline soils lengthen set time, acidic shorten.<br>• Pure gels (without sand) show higher syneresis and shrinkage; permeated sands show much greater dimensional stability.<br>• Defined silicate grouting's practical range: soil permeability between 10^{-3} m/s and 10^{-5} m/s, viscosities ≤ 20 cP.<br>• Provided design charts relating UCS, gel time, and degree of neutralization, still used as a benchmark reference in silicate grouting practice.
Elektorowicz et al. (2008) (Elektorowicz et al. 2008)	(1) Sodium silicate + ethyl acetate–formamide (SA)<br>(2) Sodium silicate + calcium chloride (SC)	Sand columns and pure gel samples, tested in freshwater and saline (NaCl, $CaCl_2$, $MgCl_2$) solutions	• SC ($CaCl_2$) grout showed superior stability, permeability remained ~10^{-6} cm/s after 60 days in both fresh and saltwater.<br>• SA (organic) grout degraded significantly, permeability rose from 10^{-7} → 10^{-4} cm/s after ~20 days in freshwater.<br>• SC formed insoluble Ca/Mg silicates, reducing leaching and strengthening the gel.<br>• Leaching results: SiO_2 ≈ 3.6 mg/L (SA, freshwater), 0.7 mg/L (SA, saline); 3.3 mg/L (SC, freshwater), 2.1 mg/L (SC, saline).<br>• pH stabilized around 8 after 6 days (SC); SA showed continuous pH rise.<br>• Carbonate film formation at 1430 cm^{-1} (IR) provided additional protection in mineralized water.
Dekker, Sweijen & Zech (2020) (Dekker et al. 2020)	Sodium silicate grout (soft gel); modeling study	Field-scale analytical and numerical modeling of a silicate grout layer	• Developed analytical solutions for groundwater flow, erosion, and mass flux through a dissolving silicate grout layer.<br>• Erosion modeled as dissolution + advection, causing time-dependent increase in hydraulic conductivity (10^{-7} → 10^{-4} m/s).<br>• Identified mass flux and dilution ratio as key metrics: μ increases with ongoing erosion → less dilution, higher contaminant release.<br>• Predicted increases in pH, Na^+, dissolved silica, and organic matter up to 10 m downstream based on prior field data.<br>• Showed erosion timescale ≈ 4 years for open pits, up to a decade when enclosed by permanent walls.
Aurand et al. (1981) (Aurand et al. 1981)	Sodium silicate grout with various hardeners: $CaCl_2$, $MgCl_2$, ethyl acetate, formamide, and sodium aluminate	Aquifer injection and groundwater chemistry	• Demonstrated that silicate gel injections alter groundwater chemistry through alkalinity increase, pH > 11, and release of soda or potash lye.<br>• Organic hardeners (e.g., ethyl acetate, formamide) introduce biodegradable organics resulting in O_2 consumption, reduction of groundwater potential, and potential sulfide precipitation of heavy metals.<br>• Inorganic hardeners ($CaCl_2$, $MgCl_2$) form insoluble Ca/Mg silicates, but raise pH and electrolyte content.<br>• Sodium aluminate produces soft gels and elevates alkalinity with minor by-products.<br>• Formamide considered problematic due to ammonia release and toxicity.

Engelhardt, Schmidt & von Borstel (2014) (HJ Engelhardt 2014)	Sodium silicate solution reacts with saline minerals (NaCl, $MgCl_2$, $CaCl_2$) in rock salt	Salt formations (evaporite rocks); laboratory & in-situ injection tests	• Demonstrated successful sealing of excavation damaged zones (EDZ) in rock salt using sodium silicate.<br>• Reaction with salts forms amorphous and crystalline Mg- and Ca-silicates, providing durable, low-solubility seals.<br>• Achieved permeability reduction to 10^{-17} m^2 in grouted salt zones.<br>• Mg^{2+} ions accelerate gelation and fix toxic metals (Co^{2+}, Ni^{2+}, Sr^{2+}, Ba^{2+}, UO_2^{2+}) into insoluble silicates, acting as a chemical barrier.<br>• Reported low syneresis rates and long-term structural stability due to formation of oxichlorides and siloxane crosslinks.<br>• Laboratory and field results show durability over long timescales (months to years) and resistance to dissolution.<br>• Concluded that sodium silicate is suitable for permanent sealing in radioactive waste repositories and salt formations due to chemical inertness and self-healing potential at saline interfaces.
Engelhardt & von Borstel (2014) (Hans-Joachim Engelhardt and Lieselotte Ernestine von Borstel	Sodium silicate reacted naturally with saline minerals (NaCl, $MgCl_2$)	Laboratory and in-situ tests in salt formations (NaCl–$MgCl_2$ brines)	• Confirmed rapid reaction between sodium silicate and rock salt, forming secondary halite and amorphous silicate phases.<br>• In $MgCl_2$-rich brines, formation of magnesium oxichlorides a stable, low-solubility compound ensuring long-term seal integrity.<br>• Demonstrated pH increase (from 7.2 to ~11) and Na^+ release during Mg^{2+} substitution.<br>• Concluded long-term chemical stability of silicate gels, even after one year of immersion tests.
Eiswirth, Ohlenbusch & Schnell (1999) (Eiswirth et al. 1999)	Sodium silicate with sodium aluminate as hardener	Urban aquifer (Berlin, Germany); field monitoring for 2 years + lab column tests	• Investigated environmental impact of ~100,000 m^3 silicate gel injected between 1990–1995 for foundation sealing.<br>• Hydrochemical monitoring of 28 wells showed increases in Na, Si, DOC only within a few meters downstream (up to ~8.5 m); beyond that, concentrations returned to background.<br>• Groundwater chemistry stabilized within 12–24 months post-injection.<br>• Modelling (PHREEQE & FEFLOW) indicated most Na^+ fixed as carbonates/aluminates, reducing mobility.<br>• Estimated leaching of Na^+ = 7–17.5% of total injected sodium mass.<br>• Concluded that silicate grout poses low long-term risk to groundwater quality; contamination confined to limited zones adjacent to grout layer.
Eiswirth & Hötzl (2003) (Eiswirth M and HÖtzel H 2003)	Soft gel sodium silicate with sodium aluminate hardener	Urban aquifer; field monitoring and lab simulation	• Investigated groundwater contamination risk following soft-gel injections used for foundation sealing.<br>• Detected elevated Na^+, SiO_2, and DOC concentrations and pH increase (to ≈11) in groundwater immediately downstream of injection sites.<br>• Elevated concentrations observed up to ~10 m downstream during the first months after injection.<br>• The plume of contamination gradually attenuated, and background levels were restored within 12–24 months.<br>• Identified that most sodium and silica were immobilized by precipitation and sorption on aquifer minerals.<br>• Concluded that soft silicate gels pose limited long-term risk to groundwater quality if injected under controlled conditions, but localized pH increases and organic leaching can occur during early stages.<br>• Recommended site-specific monitoring and careful selection of gel composition and injection pressure to minimize migration.

Malone, Barlaz & Borden (1995) (Malone et al. 1995)	Sodium silicate grout using three organic hardeners: DBE (dibasic ester mix: dimethyl glutarate, adipate, succinate), ethyl acetate, and formamide	Sand–grout mixture (Ottawa 20–30 sand)	• Evaluated leaching of organics from sodium silicate grouts prepared with three different organic hardeners.<br>• Identified reaction mechanisms: saponification (DBE, ethyl acetate) and base hydrolysis (formamide), producing sodium carboxylates, methanol/ethanol, or ammonia.<br>• ≥70% of total organic carbon (TOC) from reagents leached into solution; organics not chemically bound in gel.<br>• Formamide specimens released ~10% more organics than DBE and ~20% more than ethyl acetate; overall leachate TOC proportional to initial reagent concentration.<br>• Leaching tests (1–100 h) showed rapid initial TOC release within 1 h, with little time dependence.<br>• Concluded that organic hardeners increase potential environmental impact due to mobile, soluble byproducts (e.g., acetate, formate).<br>• Recommended future work on reagent substitution to reduce organic leachate load.

References

Ata A, Vipulanandan C (1998) Cohesive and Adhesive Properties of Silicate Grout on Grouted-Sand Behavior. Journal of Geotechnical and Geoenvironmental Engineering 124:38–44. https://doi.org/10.1061/(ASCE)1090-0241(1998)124:1(38)

Aurand K, Barowsky M, Darimont T, et al (1981) Groundwater impact of silicate gel injections. Science of The Total Environment 21:71–76. https://doi.org/10.1016/0048-9697(81)90139-X

Avci E (2017) Permeability Characteristics of Sand Grouted with Glyoxal Blended Sodium Silicate. Hittite Journal of Science and Engineering 4:71–78. https://doi.org/10.17350/HJSE19030000051

Avci E, Mollamahmutoğlu M, Deveci E (2022) Sodium silicate-formamide grouted silt and silty sand properties. J Adhes Sci Technol 36:2269–2284. https://doi.org/10.1080/01694243.2021.2010879

Berrier E, Courtheoux L, Bouazaoui M, et al (2010) Correlation between gelation time, structure and texture of low-doped silica gels. Physical Chemistry Chemical Physics 12:14477–14484. https://doi.org/10.1039/C0CP01090A

Bodocsi A, Bowers MT (1991) Permeability of acrylate, urethane, and silicate grouted sands with chemicals. Journal of Geotechnical Engineering 117:1227–1244. https://doi.org/10.1061/(ASCE)0733-9410(1991)117:8(1227)

Chen G, Xu Y, Zhang Y, Yan M (2023) Study on the differential disintegration mechanism of water glass cured sandy soil: analysis from the perspective of the coexistence state of gel and particles. Acta Geotechnica 2023 19:4 19:2191–2212. https://doi.org/10.1007/S11440-023-02002-3

Cui Y, Tan Z, Han D, Song J (2022) Investigation and application of a high performance grouting material in water-rich silty fine sand stratum. Constr Build Mater 329:127100. https://doi.org/10.1016/J.CONBUILDMAT.2022.127100

Dekker JM, Sweijen T, Zech A (2020) Groundwater flow below construction pits and erosion of temporary horizontal layers of silicate grouting. Hydrogeol J 28:2821–2832. https://doi.org/10.1007/s10040-020-02246-3

Delfosse-Ribay E, Djeran-Maigre I, Cabrillac R, Gouvenot D (2006) Factors Affecting the Creep Behavior of Grouted Sand. Journal of Geotechnical and Geoenvironmental Engineering 132:488–500. https://doi.org/10.1061/(ASCE)1090-0241(2006)132:4(488)

Dimas D, Giannopoulou I, Panias D (2009) Polymerization in sodium silicate solutions: A fundamental process in geopolymerization technology. J Mater Sci 44:3719–3730. https://doi.org/10.1007/S10853-009-3497-5/FIGURES/15

Eiswirth M, HÖtzel H (2003) Assessing the environmental risk of grouting with soft gels. RMZ - Materials and Geoenvironment 50:113–116

Eiswirth M, Ohlenbusch R, Schnell K (1999) Impact of chemical grout injection on urban groundwater. In: Impacts of Urban Growth on Surface Water and Groundwater Quality. IAHS

Elektorowicz M, Hesnawi R, Ayadat T, Chifrina R (2008) Formation of silica grout curtains and containments in mineralized groundwater. Journal of Environmental Engineering and Science 7:275–287. https://doi.org/10.1139/S08-006

Gonzalez HA, Vipulanandan C (2007) Behavior of a Sodium Silicate Grouted Sand. In: GeoDenver. American Society of Civil Engineers, pp 1–10

Gorrepati EA, Wongthahan P, Raha S, Fogler HS (2010) Silica Precipitation in Acidic Solutions: Mechanism, pH Effect, and Salt Effect. Langmuir 26:10467–10474. https://doi.org/10.1021/LA904685X

Guo S, Zhang H, Bi Y, Zhang J (2024) Mechanical experiment and microstructural characteristics of water glass solidified loess. Bulletin of Engineering Geology and the Environment 2024 83:11 83:1–21. https://doi.org/10.1007/S10064-024-03932-2

Hamouda AA, Amiri HAA (2014) Factors Affecting Alkaline Sodium Silicate Gelation for In-Depth Reservoir Profile Modification. Energie 7:568–590. https://doi.org/10.3390/EN7020568

Han T, Tan M (2012) Effects of groundwater on chemical grouting in geotechnical medium. Adv Mat Res 368–373:2848–2851. https://doi.org/10.4028/WWW.SCIENTIFIC.NET/AMR.368-373.2848

Hans-Joachim Engelhardt, Lieselotte Ernestine von Borstel (2014) The behaviour of sodium silicate solutions (water glass) in the saline environment and their use in salt mining. German J Geosci. https://doi.org/10.1127/1860-1804/2014/0057

Hashemi SJ, Hormozi F, Mokhtari R (2023) Controlling the gelation time of sodium silicate gelants for fluid management in hydrocarbon reservoirs. Fuel 341:127645. https://doi.org/10.1016/J.FUEL.2023.127645

Hatzignatiou DG, Askarinezhad R, Giske NH, Stavland A (2016) Laboratory Testing of Environmentally Friendly Sodium Silicate Systems for Water Management Through Conformance Control. SPE Production & Operations 31:337–350. https://doi.org/10.2118/173853-PA

Hatzignatiou DG, Giske NH (2018) Sodium silicate gelants for water management in naturally fractured hydrocarbon carbonate formations. Chemical Engineering Research and Design 132:40–56. https://doi.org/10.1016/J.CHERD.2017.11.041

Hatzignatiou DG, Helleren J, Stavland A (2014) Numerical Evaluation of Dynamic Core-Scale Experiments of Silicate Gels for Fluid Diversion and Flow-Zone Isolation. SPE Production & Operations 29:122–138. https://doi.org/10.2118/170240-PA

HJ Engelhardt HSL von B (2014) The Sealing of Excavation Damaged Zones in Salt Formations Using Sodium Silicate Solutions. In: DAEF Conference, Key topics in deep geological disposal. KIT Scientific Publishing

Hurley CH, Thornburn TH (1971) Sodium silicate stabilization of soils: A review of the literature. University of Illinois
James H. May, Robert J. Larson, Philip G. Malone, et al (1986) Grouting Techniques in Bottom Sealing of Hazardous Waste Sites
Kaga M, Yonekura R (1991) Estimation of Strength of Silicate-Grouted Sand. Soils and Foundations 31:43–59. https://doi.org/10.3208/SANDF1972.31.3_43
Katoueizadeh E, Rasouli M, Zebarjad SM (2021) The rheological behavior of the non-Newtonian thixotropic colloidal silica gels from sodium silicate. Mater Chem Phys 272:124994. https://doi.org/10.1016/J.MATCHEMPHYS.2021.124994
Komine H (2000) Evaluation of chemical grouted region by resistivity tomography. Proceedings of the Institution of Civil Engineers - Ground Improvement 4:177–189. https://doi.org/10.1680/GRIM.2000.4.4.177
Komine H (1997) Evaluation of chemical grouted soil by electrical resistivity. Proceedings of the Institution of Civil Engineers - Ground Improvement 1:101–113. https://doi.org/10.1680/GI.1997.010203
Krizek RJ, Spino MJ (2000) Spatial and Directional Variations in Engineering Properties of an In Situ Silicate-Grouted Sand. In: Advances in Grouting and Ground Modification. American Society of Civil Engineers, pp 139–154
LIAN X, PENG Z hong, SHEN L ting, et al (2021) Properties of low-modulus sodium silicate solution in alkali system. Transactions of Nonferrous Metals Society of China 31:3918–3928. https://doi.org/10.1016/S1003-6326(21)65774-6
Littlejohn G, Concannon M, Wright R (1997) ENGINEERING PROPERTIES OF SILICATE-R100 ESTER CHEMICAL GROUTS. Ground engineering
Littlejohn GS, Mollamahmutoglu M (1994) Time-dependent behaviour of silicate grouted sand. Grouting in the ground 37–51. https://doi.org/10.1680/GITG.19287.0004
Malone J, Barlaz M, Borden R (1995) Leachability of Organic Compounds from Sodium Silicate Grouts Containing Organic Reagents. In: Twenty-Seventh Mid-Atlantic Industrial Waste Conference. CRC Press, pp 460–469
Matinfar M, Elias A, Nychka JA (2025) Microstructure and mechanical properties of silica hydrogels from sodium silicate solutions. Mater Des 253:113919. https://doi.org/10.1016/J.MATDES.2025.113919
Matinfar M, Nychka JA (2023) A review of sodium silicate solutions: Structure, gelation, and syneresis. Adv Colloid Interface Sci 322:103036. https://doi.org/10.1016/J.CIS.2023.103036
Matinfar M, Nychka JA (2025) Molecular structure determination of acid-initiated sodium silicate sol-gels via Raman spectroscopy. J Non Cryst Solids 666:123671. https://doi.org/10.1016/J.JNONCRYSOL.2025.123671
Matinfar M, Nychka JA (2024) Process Mapping of the Sol–Gel Transition in Acid-Initiated Sodium Silicate Solutions. Gels 10:673. https://doi.org/10.3390/GELS10100673
Mollamahmutoglu M (1999) Effect of incremental loading on the creep behaviour of chemically grouted sand. Bulletin of Engineering Geology and the Environment 57:353–358. https://doi.org/10.1007/S100640050058/METRICS
Mollamahmutoğlu M, Avci E (2020) Effects of Particle Gradation, Relative Density and Curing on the Strength of Silicate Grouted Sand. Geotechnical and Geological Engineering 38:6695–6715. https://doi.org/10.1007/S10706-020-01463-7
Mollamahmutoğlu M, Avci E (2016) Syneresis effect on the permeability of chemically grouted sand. Quarterly Journal of Engineering Geology and Hydrogeology 49:327–335. https://doi.org/10.1144/qjegh2015-104
Mollamahmutoğlu M, Avci E, Tomaç SK, Köse DA (2017) Performance of Novel Chemical Grout in Treating Sands. Journal of Materials in Civil Engineering 29:. https://doi.org/10.1061/(ASCE)MT.1943-5533.0002004
Mollamahmutoğlu M, Avcı E, Deveci E, et al (2021) Strength and permeability properties of sodium silicate-sodium dihydrogen phosphate grouted sand. Arabian Journal of Geosciences 14:. https://doi.org/10.1007/S12517-021-08427-Y
Mollamahmutoğlu M., Littlejohn G (1995) A review of some of the properties of Geoseal MQ-5 and silicate-hardener 600B grouts. GROUND ENGINEERING 28:44–48
Mollamahmutoglu M, Littlejohn S (1997) Varying temperature and creep of silicate grouted sand. Proceedings of the Institution of Civil Engineers - Ground Improvement 1:59–64. https://doi.org/10.1680/GI.1997.010106
Nasr-El-Din HA, Taylor KC (2005) Evaluation of sodium silicate/urea gels used for water shut-off treatments. J Pet Sci Eng 48:141–160. https://doi.org/10.1016/J.PETROL.2005.06.010
Pham HG, Nguyen QC, Meng HL (2011) Monitoring the Chemical Grouting in Sandy Soil by Electrical Resistivity Tomography (ERT). In: 1st International Workshop on Geoelectrical Monitoring. Applications in Engineering, Vienna
Pham LT, Hatzignatiou DG (2016) Rheological evaluation of a sodium silicate gel system for water management in mature, naturally-fractured oilfields. J Pet Sci Eng 138:218–233. https://doi.org/10.1016/J.PETROL.2015.11.039
Porcino D, Marcianò V, Granata R (2012) Static and dynamic properties of a lightly cemented silicate-grouted sand. https://doi.org/101139/t2012-069 49:1117–1133. https://doi.org/10.1139/T2012-069
Quarch K, Kind M (2010) Inorganic Precipitated Silica Gel. Part 1: Gelation Kinetics and Gel Properties. Chem Eng Technol 33:1034–1039. https://doi.org/10.1002/CEAT.201000080
Salehzadeh H, Hassanlourad M, Shahnazari H (2012) Shear behavior of chemically grouted carbonate sands. International Journal of Geotechnical Engineering 6:445–454. https://doi.org/10.3328/IJGE.2012.06.04.445-454
Tognonvi MT, Lecomte A, Rossignol S, Bonnet JP (2019) Ripening of Na-silicate gels in basic media: Identification of involved chemical species. J Non Cryst Solids 522:119482. https://doi.org/10.1016/J.JNONCRYSOL.2019.119482
Tognonvi MT, Rossignol S, Bonnet JP (2011) Physical-chemistry of sodium silicate gelation in an alkaline medium. J Solgel Sci Technol 58:625–635. https://doi.org/10.1007/S10971-011-2437-4
US Army Corps of Engineers (1995) Engineering and Design: CHEMICAL GROUTING (EM 1110-1-3500). Department of the Army, Washington
Vipulanandan C, Ata A (2000) Cyclic and Damping Properties of Silicate-Grouted Sand. Journal of Geotechnical and Geoenvironmental Engineering 126:650–656. https://doi.org/10.1061/(ASCE)1090-0241(2000)126:7(650)
Vipulanandan C, Krizek RJ (1986) Mechanical Behavior of Chemically Grouted Sand. Journal of Geotechnical Engineering 112:869–887. https://doi.org/10.1061/(ASCE)0733-9410(1986)112:9(869)
Visser JHM (2018) Fundamentals of alkali-silica gel formation and swelling: Condensation under influence of dissolved salts. Cem Concr Res 105:18–30. https://doi.org/10.1016/J.CEMCONRES.2017.11.006
Wang X, Wang C, Li P, et al (2023) Experimental study on new grouting material of acidic sodium silicate and its properties of grouted-sand. Constr Build Mater 392:131955. https://doi.org/10.1016/J.CONBUILDMAT.2023.131955
Wijnen PWJG, Beelen TPM, de Haan JW, et al (1989) Silica gel dissolution in aqueous alkali metal hydroxides studied by 29Si□NMR. J Non Cryst Solids 109:85–94. https://doi.org/10.1016/0022-3093(89)90446-8
Wilhelm S, Kind M (2014) On the Relation between Natural and Enforced Syneresis of Acidic Precipitated Silica. Polymers (Basel) 6:2896–2911. https://doi.org/10.3390/POLYM6122896
Wilhelm S, Kind M (2015) Influence of pH, Temperature and Sample Size on Natural and Enforced Syneresis of Precipitated Silica. Polymers (Basel) 7:2504–2521. https://doi.org/10.3390/POLYM7121528

Xu Y, Wei T, Chen G, et al (2023) Experimental study on the development mode and evolution mechanism of sodium silicate solidified sand under different temperatures and curing paths. Constr Build Mater 409:134073. https://doi.org/10.1016/J.CONBUILDMAT.2023.134073
Xu Y, Zhang Y, Huang J, Chen G (2022) Mechanical properties, microstructure and consolidation of sand modified with sodium silicate. Eng Geol 310:106875. https://doi.org/10.1016/J.ENGGEO.2022.106875
